# Bone marrow mesenchymal stem cells and their derived exosomes resolve doxorubicin-induced chemobrain: critical role of their miRNA cargo

**DOI:** 10.1186/s13287-021-02384-9

**Published:** 2021-06-05

**Authors:** Marwa O. El-Derany, Mohamed H. Noureldein

**Affiliations:** 1 Department of Biochemistry, Faculty of Pharmacy, Ain Shams University, Cairo, 11566 Egypt; 2 Department of Anatomy, Cell Biology and Physiological Sciences, Faculty of Medicine, American University of Beirut, Beirut, Lebanon; 3 American University of Beirut Diabetes Program, Beirut, Lebanon

**Keywords:** Chemobrain, BMSCs, Exosomes, miRNAs, Signaling pathway

## Abstract

**Background:**

Doxorubicin (DOX), a widely used chemotherapeutic agent, can cause neurodegeneration in the brain, which leads to a condition known as chemobrain. In fact, chemobrain is a deteriorating condition which adversely affects the lives of cancer survivors. This study aimed to examine the potential therapeutic effects of bone marrow mesenchymal stem cells (BMSCs) and their derived exosomes (BMSCs-Exo) in DOX-induced chemobrain in rat models.

**Methods:**

Chemobrain was induced by exposing rats to DOX (2 mg/kg, i.p) once weekly for 4 consecutive weeks. After 48 h of the last DOX dose, a subset of rats was supplied with either an intravenous injection of BMSCs (1 × 10^6^) or a single dose of 150 μg of BMSCs-Exo. Behavioral tests were conducted 7 days post injection. Rats were sacrificed after 14 days from BMSCs or BMSCs-Exo injection.

**Results:**

BMSCs and BMSCs-Exo successfully restored DOX-induced cognitive and behavioral distortion. These actions were mediated via decreasing hippocampal neurodegeneration and neural demyelination through upregulating neural myelination factors (myelin%, Olig2, Opalin expression), neurotropic growth factors (BDNF, FGF-2), synaptic factors (synaptophysin), and fractalkine receptor expression (Cx3cr1). Halting neurodegeneration in DOX-induced chemobrain was achieved through epigenetic induction of key factors in Wnt/β-catenin and hedgehog signaling pathways mediated primarily by the most abundant secreted exosomal miRNAs (miR-21-5p, miR-125b-5p, miR-199a-3p, miR-24-3p, let-7a-5p). Moreover, BMSCs and BMSCs-Exo significantly abrogate the inflammatory state (IL-6, TNF-α), apoptotic state (BAX/Bcl2), astrocyte, and microglia activation (GFAP, IBA-1) in DOX-induced chemobrain with a significant increase in the antioxidant mediators (GSH, GPx, SOD activity).

**Conclusions:**

BMSCs and their derived exosomes offer neuroprotection against DOX-induced chemobrain via genetic and epigenetic abrogation of hippocampal neurodegeneration through modulating Wnt/β-catenin and hedgehog signaling pathways and through reducing inflammatory, apoptotic, and oxidative stress state.

**Graphical abstract:**

Proposed mechanisms of the protective effects of bone marrow stem cells (BMSCs) and their exosomes (BMSCs-Exo) in doxorubicin (DOX)-induced chemobrain. Blue arrows: induce. Red arrows: inhibit. 
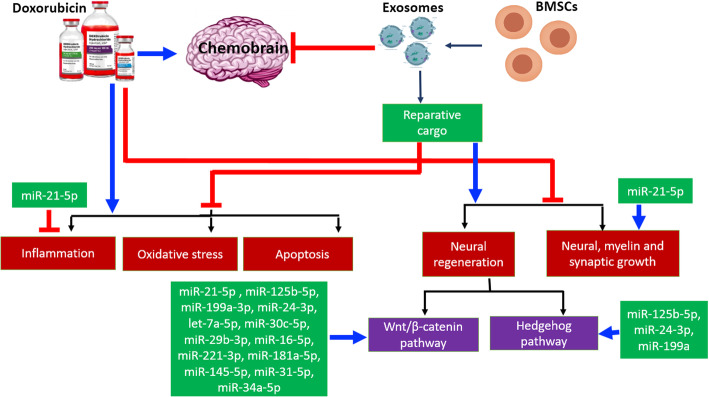

**Supplementary Information:**

The online version contains supplementary material available at 10.1186/s13287-021-02384-9.

## Introduction

Given that brain neurons are highly vulnerable to environmental toxins, it is not surprising that they are destructively injured by cytotoxic drugs [1]. Particularly, doxorubicin (DOX), a natural anthracycline antibiotic, has long been considered as a standard cornerstone chemotherapeutic agent for numerous types of cancer especially breast cancer; however, its use has been hampered by its toxic side effects especially on the brain [2]. These side effects cause decline in memory, concentration, educational attainment, and executive functions which can last for up to several years causing significant distress among patients [3]. These cognitive side effects of chemotherapy are referred to as chemobrain or chemofog. Imaging and functional studies confirmed structural and functional alterations in the brain plasticity, axonal demyelination, reduction of hippocampal neurogenesis, as well as changes in growth factor levels among DOX users [4]. In this context, DOX has been marked as a chemobrain inducer through altered cytokine paradigm. Being an indispensable chemotherapeutic drug, numerous researches have been directed to ameliorate the overwhelming DOX-induced cognitive side effects.

Restoration of neural damage is not an easy task, with no pharmacological therapy being approved yet. Nevertheless, advances in regenerative medicine have identified mesenchymal stem cells (MSCs) as a potential cell therapy for the brain repair in multiple neurodegenerative diseases such as Parkinson’s disease, Alzheimer’s, and stroke [5]. Specifically, bone marrow mesenchymal stem cells (BMSCs), apart from being easily isolated and cultured, display favorable proliferative profile with reduced immunological reaction. In view of these prospects, BMSCs have received much attention in multiple neurological disorders [6, 7].

Notably, promising reports showed that BMSCs secrete extracellular vesicles known as exosomes containing a reparative cargo with various miRNA, neurotrophic factors, and cytokines which impose significant anti-inflammatory and anti-apoptotic effects. With well-recognized immunomodulatory properties, BMSCs and BMSC-derived exosomes (BMSCs-Exo) can modulate microglia and astrocyte reactivity, thereby promoting neuro-regeneration [8, 9]. However, identifying the molecular and cellular machinery that impact neural microenvironment after stem cell therapy is still a necessity.

Neurodegeneration is promoted by disruption of multiple signaling pathways controlling neurogenesis in the context of aggravated inflammatory and oxidative stress states [10]. Recent studies highlighted the canonical crosstalk between Wnt/β-catenin and hedgehog signaling pathways to facilitate the dynamic modulation of neurogenesis [11]. In the same line, these signaling pathways are reported to be disrupted in multiple neurodegenerative diseases [12, 13]. Besides, these pathways were found to be intricately associated with resistance to DOX treatment [14]. Thus, their modulation could have a synergistic effect for cancer treatment [15, 16] and might prevent neurodegeneration associated with DOX administration.

Interestingly, stem cells are known to distinctively regulate these integrative pathways in number of diseases [17–19]. Nevertheless, studying the impact of stem cells on these integrated signaling pathways in DOX-induced chemobrain needs to be elucidated.

Accordingly, this study aimed to mechanistically study the therapeutic effects of BMSCs and BMSCs-Exo in DOX-induced chemobrain. We aimed to determine the behavioral, histopathological improvements associated with BMSCs or BMSCs-Exo treatment. Besides, this study aimed to scrutinize the underlying signaling crosstalk in DOX-induced chemobrain pathogenesis and treatments by BMSCs and BMSCs-Exo.

## Materials and methods

### Isolation of BMSCs and BMSCs-Exo

BMSCs were isolated from the tibia and femurs of 4-week-old healthy albino rats as previously described [20]. Briefly, whole bone marrow was aspirated and bones were flushed with low-glucose Dulbecco’s modified Eagle’s medium (LDMEM) supplemented with 10% bovine serum (Lonza, USA), 100 U/mL penicillin, and 100 μg/mL streptomycin (Gibco-BRL, Grand Island, NY, USA). The cells were seeded into culture vessels and cultured in a humidified incubator at 37 °C with 5% CO_2_. The medium was replaced after 72 h of culture, the non-adherent cells were removed, and adherent cells were recognized as BMSCs. Cells are recognized by uniform morphological appearance as fibroblast-like long spindles. Cell number and viability were evaluated with trypan blue using a hemocytometer device (Invitrogen, USA). Cells were detected under an inverted phase-contrast microscope (Olympus, USA).

To extract exosomes secreted by BMSCs, cells were cultured in serum-free media. Then the conditioned media of BMSCs was collected and centrifuged at 2000×*g* at 4 °C for 10 min, followed by 10,000×*g* at 4 °C for 30 min to remove cell debris. The supernatant was then ultracentrifuged at 100,000×*g* for 70 min to pellet the exosomes. Exosomes were washed with PBS and then ultracentrifuged at 100,000×*g* for 70 min (Thermo Scientific, USA). Isolated exosomes were resuspended in 150 μL of particle-free PBS.

### Characterization of BMSCs and BMSCs-Exo

Cell surface marker expression was analyzed for cells at passage 4 (P4) as previously described [21]. The cells were stained for 30 min with FITC-conjugated anti-rat CD105 (R&D, FAB10971F), PE-conjugated anti-rat CD73 (R&D, FAB5796P), PE-conjugated anti-rat CD34 (Beckman coulter, IM3630A), antibodies, and PE-conjugated anti-rat CD14 (Beckman coulter, IM0650U) antibodies (Beckman Coulter, Brea, CA, USA). For gating, unstained cells were used as controls. Expression of exosome surface markers was analyzed for BMSCs-Exo as previously described using PE-conjugated anti-mouse CD63 (BioLegend, San Diego, CA). Analysis was performed using a CYTOMICS FC 500 Flow Cytometer (Beckman Coulter, Brea, CA, USA) and analyzed using CXP Software version 2.2. Additionally, analysis of the exosomes using transmission electron microscopy (TEM) was performed after suspending the exosomes in PBS buffer. This suspension was applied onto a carbon-coated copper grid, followed by staining with 2% uranyl acetate. Images of exosomes were obtained using an electron microscope (JEM-1010; JEOL Ltd., Tokyo, Japan) at an acceleration voltage of 70 kV.

### Multipotent differentiation of BMSCs

Adipogenic and osteogenic differentiation were performed by culturing BMSCs in adipogenic and osteogenic induction medium (StemXVivo®, R&D Systems) for 21 and 14 days respectively. Oil droplets were recognized by oil red staining and calcium deposition was authenticated by Alizarin Red staining. Staining was detected under an inverted phase-contrast microscope (Olympus, USA).

### Labeling of BMSCs with PKH26

BMSCs were collected after P4 and labeled with PKH26 Red Fluorescent Cell Linker kit (Sigma-Aldrich, USA), according to the manufacturer’s instructions. Four female albino rats were purchased and received DOX hydrochloride (Sigma-Aldrich, St. Louis, MO, USA) dissolved in 0.9% sodium chloride once weekly in a dose of 2 mg/kg, intra-peritoneal (i.p.) for 4 consecutive weeks. Forty-eight hours after the last DOX dose, labeled 1 × 10^6^ BMSCs per rat were intravenously injected into the tail vein. After 24 h, rats were anesthetized with ketamine (100 mg/kg, i.p.) and then sacrificed by cervical dislocation and the whole brains were excised. Specific fluorescence versus brain tissue background was analyzed using a Leica microscope (excitation 490 nm/emission 570 nm) to detect and trace the labeled stem cells.

### In vivo experiments

Female albino rats weighing 150 g were purchased from the animal house facility, National Research Center (Giza, Egypt). The animals were housed in stainless-steel cages in air-conditioned chamber (24 ± 2 °C) with alternating 12 h day/night cycles. Animals were allowed access to standardized food pellets and water ad libitum and left for 1 week to acclimatize before starting the experiment. The experimental protocol was carried out in accordance with the Guide for Care and Use of Laboratory Animals published by the US National Institutes of Health (NIH Publication No. 85-23, revised 2011) and was approved by the Research Ethics Committee, Faculty of Pharmacy, Ain Shams University, Cairo, Egypt. Rats were randomly assigned into four groups (n = 20/group) and treated for 4 weeks as follows:

The first group served as the control group and received i.p injection of 0.9% sodium chloride given once weekly for 4 consecutive weeks. Forty-eight hours later, a single intravenous injection of 150 μL of particle-free PBS was given.

The second group served as DOX-treated group and received DOX hydrochloride dissolved in 0.9% sodium chloride given once weekly in a dose of 2 mg/kg, i.p. for 4 consecutive weeks. Forty-eight hours later, a single intravenous injection of 150 μL of particle-free PBS was given. DOX was administered as previously described [22], following a schedule similar to that used in patients with breast cancer. Whereas in alignment with previous studies, this dose is believed to cause hippocampal-based memory deficits and severe disruptions of hippocampal neurogenesis in a rat model of chemobrain [22–25].

The third group received DOX once weekly (2 mg/kg, i.p.) for 4 consecutive weeks followed by a single intravenous injection of (1 × 10^6^) BMSCs that was given 48 h after the last DOX dose. This dose range was chosen in accordance with doses used in various in vivo studies of BMSCs in different cognitive impairment diseases [26–29].

**Fig. 1:**
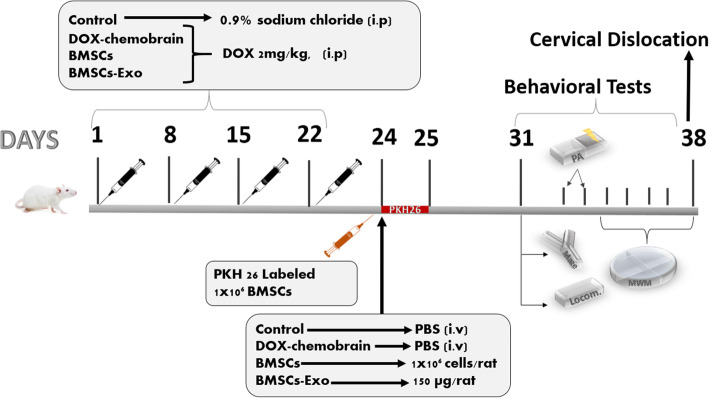
An illustration of the study design showing the timeline of the induction of chemobrain by doxorubicin (DOX) and the treatment with bone marrow stem cells (BMSCs) or their exosomes (BMSCs-Exo), and behavioral test schedule. Rats were randomly assigned into four groups (n = 20/group) and treated for 4 weeks as follows: The first group served as the control group and received intra-peritoneal (i.p) injection of 0.9% sodium chloride given once weekly for 4 consecutive weeks. Forty-eight hours later, a single intravenous injection of 150 μL of particle-free PBS was given. The second group served as DOX-treated group and received DOX hydrochloride dissolved in 0.9% sodium chloride and given once weekly in a dose of 2 mg/kg, i.p. for 4 consecutive weeks. Forty-eight hours later, a single intravenous injection of 150 μL of particle-free PBS was given. The third group received DOX once weekly (2 mg/kg, i.p.) for 4 consecutive weeks followed by a single intravenous injections of (1 × 10^6^) BMSCs per rat that was given 48 h after the last DOX dose. The fourth group was the BMSCs-Exo-treated group and received DOX once weekly (2 mg/kg, i.p.) for 4 consecutive weeks followed by a single dose of 150 μg of exosomal proteins per rat that was given 48 h after last DOX dose. Four rats were treated by labeled PKH26 Red Fluorescent Cell Linker BMSCs, and after 24 h, rats were then anesthetized with ketamine (100 mg/kg, i.p.) and sacrificed by cervical dislocation the whole brains were excised. Behavioral testing started 7 days after BMSCs and BMSCs-Exo administration. After 14 days from BMSCs and BMSCs-Exo injection, the four groups were then anesthetized with ketamine (100 mg/kg, i.p.) and sacrificed by cervical dislocation, the whole brains were excised, and hippocampi were dissected and weighed

The fourth group was the BMSCs-Exo-treated group and received DOX once weekly (2 mg/kg, i.p.) for 4 consecutive weeks followed by a single dose of 150 μg per rat of exosomal proteins (approximate amount produced by 6 × 10^6^ BMSCs) that was given 48 h after last DOX dose. This dose was chosen in accordance with previous studies of MSC-derived exosomes in different cognitive impairment diseases [30]. Drugs, BMSCs and BMSCs-Exo administration, behavioral tests, and sacrifice were conducted as shown in the timeline (Fig. 1).

Behavioral testing started 7 days after BMSCs and BMSCs-Exo administration where the rats were transferred to the behavioral lab in their home cages to acclimatize before starting the behavioral tests. Behavioral tests were performed by experimenters that were blind to the treatment conditions. Behavioral tests were conducted on all groups, including the control groups. Rats were then anesthetized with ketamine (100 mg/kg, i.p.) and sacrificed by cervical dislocation after 14 days from BMSCs and BMSCs-Exo injections and the whole brains were excised, and hippocampi were dissected and weighed. Specimens were either stored at − 80 °C for neurochemical analyses or were fixed in 10% formalin for the preparation of paraffin blocks for either histopathological or immunohistochemical assessment.

### Behavioral tests

#### Memory retention by step-through passive avoidance

Step-through passive avoidance (PA) apparatus (UgoBasile, Comerio, Italy) is divided into two chambers: white lighted chamber and a black dark one. The grid floor of the dark chamber can be programmed to deliver an electric shock of the required intensity whenever stepped on. The two chambers are separated by an automatically operated sliding gate. Each rat was subjected to two sessions; acquisition session to acclimatize (training) in the first day and retention session (test) after 24 h from training. During the training session, rats were gently placed individually in the lighted chamber. When a rat stepped through the dark compartment, placing its 4 paws on the grid floor, the sliding door closed, and an electric shock of 1 mA was delivered for 2 s. Twenty-four hours later, rats were re-placed gently in the light chamber and their latency to step through the dark chamber was recorded and considered as a passive avoidance behavior to evaluate their memory acquisition after being exposed to an electrical shock. This test evaluates the contextual fear for assessing memory changes. The cut-off time was set to 3 min in both the training and the retention sessions (i.e., both were of equal total time); only one trial for each rat was included in each of the acquisition and retention sessions (one trial each day). Besides, no electric shocks were delivered during test sessions [23].

#### Morris water maze test (MWM)

The apparatus consisted of a circular water pool (120 cm diameter, 60 cm in height), containing water to a depth of 15.5 cm. The water temperature was maintained at 24 ± 1 °C and was rendered opaque by addition of milk powder. The pool was virtually divided into four quadrants, i.e., North (N), South (S), East (E), and West (W). A transparent platform (10 cm diameter) was hidden 1.5 cm below the surface of water and placed at the midpoint of the fourth quadrant “SW.” The test was conducted as previously described [31, 32]. The test trial ends by either finding the platform or continuing for a maximum of 90 s. Briefly, each rat was trained to acclimatize for 4 consecutive days. Each rat was given a series of daily trials using a semi-random set of start locations. Semi-random start position sets were used such that the four positions are used, with the restriction that one trial each day for each of the four positions (each of the four start positions were used once each day) which were described previously [32]. During the first four training days, the rats were placed into the maze pool to reach the hidden platform using four semi-random set of start locations as mentioned previously (day 1: N, E, SE, NW), (day 2: SE, N, NW, E), (day 3: NW, SE, E, N), and (day 4: E, NW, N, SE). These set of start locations are designed so that rats will not be able to learn a specific order of right or left turns to locate the platform [32]. The time allowed for the rats to reach the platform is 60 s, then, rats were allowed to sit on the platform for 30 s. Those who failed to find the platform in 60 s were guided to the platform and could sit on the platform for 30 s. Each rat was subjected to four trials every day for four consecutive days as previously described [33].

On the fifth day, a probe trial was performed to evaluate the extent of memory consolidation as previously reported [34]. On the fifth day, the platform was removed, and the rats were placed and released at (NE) opposite to the site where the platform had been located (SW). The single trial consisted of a 90-s swim in the pool without the platform. The time spent in the target quadrant indicated the degree of memory consolidation after learning and the percentage of time spent in the former platform was calculated for the probe trial. All data were recorded with a video system.

#### Short-term spatial memory evaluation

The Y-maze apparatus consists of a black wood maze with 3 similar opaque arms (40 cm length, 15 cm height, and 8 cm width) intersected at 120° and were labeled as A, B, or C. The animal is positioned in the start arm B and permitted to acclimatized and explore the 3 arms for 5 min. Afterwards, rats were put at the starting area to begin the experiment. A spontaneous alternation was recorded for 5 min and counts begin when 4 paws of the rat are inside the arm and the rat had entered the three different arms sequentially. Spontaneous alteration behavior was defined as the entry into all three arms on consecutive choices in overlapping triplet sets (e.g., ABC, BCA, CAB) [35].

The total number of alternations and total arm entries (TAE) were documented, and the spontaneous alternation percentage (SAP) was computed from it according to the following formula: “the number of alternations” divided by “the total possible alternations (i.e., the total number of arm entries minus 2)” and multiplied by 100, i.e., SAP = [(number of alternations)/(TAE − 2)] × 100 [36]. Pearson’s correlation analysis [37] was performed of SAP to TAE made, to exclude the influence of hyper- or hypodynamic locomotion on the apparent cognitive endpoint [38].

#### Locomotor activity assessment

Activity monitor (Opto-Varimex-Mini Model B, Columbus Instruments, OH, USA) was used to evaluate the locomotor activity of animals based on the traditional infrared photocell principle (68 × 68 × 45 cm) equipped with 15 infrared (IR) beams (wavelength = 875 nm and diameter = 0.32 cm), spaced 2.65 cm apart, and scan rate = 160 Hz. The principle of measurement depends on the emittance of evenly spaced infrared light beams, where beam interruptions caused by movements of the animal are sensed and counted. Prior to starting the test, each rat was gently placed in the activity monitor chamber for 5 min to acclimatize. Then, locomotion of each animal was calculated as the number of movements per 5 min [39].

### Histopathological examination, neural, and myelin staining

Four brain tissue samples were taken from different groups and fixed in 10% neutral buffered formalin for 72 h. Samples were trimmed and processed in serial grades of alcohols, and cleared in Xylene. Subsequently, samples were infiltrated and embedded into paraplast tissue embedding media where paraffin tissue blocks were thus prepared. Four-micrometer-thick sagittal brain sections were cut by rotatory microtome for demonstration of hippocampal regions in different samples. The obtained tissue sections were collected on glass slides and stained by hematoxylin and eosin stains as a general morphological examination staining method and examined by a light microscope (Leica Microsystems GmbH, Wetzlar, Germany) as previously described [40].

As previously described, tissues were also stained by toluidine blue stain for demonstration of damaged and intact neurons and examined by using a light microscope (Leica Microsystems GmbH, Wetzlar, Germany) [41]. Six non-overlapping fields were randomly selected and scanned by experienced histologist from CA1 and CA3 hippocampal subregions per tissue section to determine the numbers of intact neurons counts in Toluidine blue-stained tissue sections in each region in four samples in each group.

Additionally, tissues were also stained by Luxol fast blue stain for demonstrating the myelinated nerve fibers in corpus callosum regions and examined by using a light microscope (Leica Microsystems GmbH, Wetzlar, Germany). Six non-overlapping fields were randomly selected and scanned from corpus callosum regions for quantification of positively stained mylinated nerve fibers to calculate the total mean expression levels of four samples in each group.

### Immunohistochemical analysis

From the previously prepared embedded tissue blocks, 5-μm-thick paraffin section were cut by rotatory microtome for demonstrating immune reactions in hippocampal regions in different samples. Immunohistochemical staining was conducted according to the manufacturer’s protocol. Deparaffinized tissue sections were treated by 3% H_2_O_2_ for 20 min, washed out by PBS. Followed by incubation with different antibodies to detect presynaptic protein synaptophysin (SY 38), β-catenin, glial fibrillary acidic protein (GFAP), and ionized calcium-binding adaptor molecule 1 (IBA-1) using ready to-use primary antibodies; monoclonal antibody to rat SY38 (DAKO, Cat# M0776), rabbit polyclonal antibody to rat β-catenin (Abcam, Ab16051), mouse monoclonal antibody to GFAP (Thermo Scientific Co., Cat. No. MS-280-P1), and IBA-1 antibody (Abcam, ab108539) at 4 °C overnight. Each was subsequently washed out by PBS, followed by incubation with secondary antibody HRP Envision kit (DAKO) for 20 min, then washed by PBS and incubated with diaminobenzidine (DAB) for 15 min followed by another wash with PBS then counterstaining with hematoxylin, dehydration in xylene then covered for microscopic examination as previously described [42]. Immunohistochemical data was represented as mean expression levels for total scanned hippocampal region with at least six random non-overlapping fields. They were scanned and segmented for each subregion by the same histologist to calculate the total mean expression levels in each section of immunostained samples in automated manner by using Leica application module for area-based quantitative analysis of four samples in each group. Morphological measurements and analyzed data were obtained using Leica Application module for tissue sections analysis attached to full HD microscopic imaging system (Leica Microsystems GmbH, Germany) operated by Leica Application software for tissue sections analysis.

### Reverse transcription-quantitative real-time polymerase chain reaction (RT-qPCR)

RT-qPCR was used to determine the gene expression of neural myelination genes such as oligodendrocyte transcription factor-2 (Olig2) and oligodendrocytic paranodal loop protein (Opalin) genes, neural and synaptic growth factors such as fibroblast growth factor-2 (FGF-2) and Synaptophysin (Syp) genes, neurogenesis signaling, and transcription factors genes such as Shh, patched-1 (Ptch-1), glioma-associated oncogene family zinc finger (Gli), wingless-type MMTV integration site family (Wnt), frizzled (FZD), beta-catenin (β-catenin), neurogenic differentiation 1 (Neurod1), prospero-related homeobox (Prox1), Fox-3, Rbfox3, hexaribonucleotide binding protein-3 (NeuN), doublecortin (DCX), sex-determining region Y-box 2 (SOX2), CX3C chemokine receptor 1 (Cx3cr1) (fraktalkine receptor), apoptotic genes such as B cell lymphoma 2 (Bcl2), Bcl2-associated X (Bax) genes. Total RNA was extracted from the hippocampus using Qiagen tissue extraction Kit (Qiagen, USA) and reversely transcribed using high-capacity cDNA Synthesis Kit (Thermo Scientific Co., USA). RT-qPCR was performed using an ABI 7500 RT-PCR System (Applied Biosystems, Foster City, CA, USA) and powerup SYBR® Green PCR Master Mix (Thermo Scientific co., USA) (Applied Biosystems). Sequences of PCR primer pairs used for all genes as well as the reference control β-actin gene (Thermo Scientific Co., USA) are shown in Table 1.

**Table 1:** Sequences of primers sets used for gene expression analysis

Gene symbol	Primer sequence	GenBank accession number
Olig2 F: Olig2 R:	5′- TTCAGACCACATGAGCAAGC-3′ 5′- TAAAAACAGCGTCCCCAGTC-3′	NM_001100557.1
Opalin F: Opalin R:	5'-AAGTGTATCCCAGCTTGCCT-3' 5'-CTTCCCGATGTCTCTCCCTC-3'	NM_001017386.1
FGF-2 F: FGF-2 R:	5’-CCCACACGTCAAACTACAGC-3' 5'-CTGCCCAGTTCGTTTCAGTG-3'	NM_019305.2
Syp F: Syp R:	5′-ACAGGAAGGGAACCAGACCT-3′ 5′-CAAGCCTCCTCCACTCAGTC-3′	NM_012664.3
Shh F: Shh R:	5′- CAATTACAACCCCGACATCA-3′ 5′-AGTCACTCGAAGCTTCACTCC-3′	NM_017221.1
Ptch-1 F: Ptch-1 R:	5′-ACGCTCCTTTCCTCTTGAGAC-3′ 5′-TGAACTGGGCAGCTATGAAGT C-3′	NM_053566.1
Gli-1 F: Gli-1 R:	5′-CAGGGAAGAGAGCAGACTGAC -3′ 5′-CAGGAGGATTGTGCTCCA -3′	NM_001191910.1
WNT2 F: WNT2 R:	5′- AGGCAGCGTTTGTCTATGCT-3′ 5′- GTCACTACAGCCACCCCAGT-3′	XM_575397.7
WNT3a F: WNT3a R:	5′- CCTGGTTGGGGTCAGTAAGA-3′ 5′- GGTAGAGAGTGCAGGCAAGG-3′	NM_001107005.2
FZD1 F: FZD1 R:	5′- GCGACGTACTGAGCGGAGTG-3′ 5′- TGATGGTGCGGATGCGGAAG-3′	NM_021266.3
Neurod1 F: Neurod1 R:	5′- ACAGCTCCCATGTCTTCCAC-3′ 5′- AAGATTGATCCGTGGCTTTG-3′	NM_019218.2
Prox1 F: Prox1 R:	5′- GAAGGGCTATCACCCAATCA-3′ 5′- TGGACAGTTCCTCTGTGCTG-3′	NM_001107201.1
NeuN F: NeuN R:	5'-TTGCTTCCAGGGTCGTGTAT-3' 5'-GGGCCGATGGTATGATGGTA-3'	NM_001134498.2
DCX F: DCX R:	5′- CCTTCAATGTCATGGCACAC-3′ 5′- CTCTGGCTTGGCTCACTACC-3′	NM_053379.3
Sox2 F: Sox2 R:	5'-ATTACCCGCAGCAAAATGAC-3' 5'-ATCGCCCGGAGTCTAGTTCT-3'	NM_001109181.1
Cx3cr1 F: Cx3cr1 R:	5'-CCAACTCCATGAACAACCGG-3' 5'-GATGTTGACCTCCGAGTTGC-3'	NM_133534.1
Bax F: Bax R:	5′-GATCAGCTCGGGCACTTTA-3′ 5′-TGTTTGCTGATGGCAA CTTC-3′	NM_017059.2
Bcl2 F: Bcl2 R:	5′-AGGAT TGTGG CCTTC TTTGA GT-3′ 5′-GCCG GTTC AGG TACT CAGT CAT-3′	NM_016993.1
β-actin F: β-actin R:	5′- TGTCACCAACTGGGACGATA-3′ 5′- GGGGTGTTGAAGGTCTCAAA-3′	NM_031144.3

*Olig2*, oligodendrocyte transcription factor-2; *Opalin*, oligodendrocytic paranodal loop protein; *FGF-2*, fibroblast growth factor-2; *Syp*, Synaptophysin; *Shh*, Sonic hedgehog; *Ptch-1*, patched-1; *Gli*, glioma-associated oncogene family zinc finger; *Wnt*, wingless-type MMTV integration site family; *FZD1*, frizzled1; *Neurod1*, neurogenic differentiation 1; *Prox1*, prospero-related homeobox; *NeuN*, hexaribonucleotide binding protein-3; *DCX*, doublecortin; *SOX2*, sex-determining region Y-box 2; *β-actin*, beta-actin; *Cx3cr1*, CX3C chemokine receptor 1; *Bcl2*, B cell lymphoma 2; *BAX*, BCL2-associated X

Data were analyzed with ABI Prism sequence detection software and quantified using the v1.7 Sequence Detection Software from PE Biosystems (Applied Biosystems, Foster City, CA). Relative expression of studied genes was calculated using the comparative threshold cycle method. All values were normalized to β-actin gene as an invariant endogenous control (reference gene). The relative quantification was then calculated by the 2^−ΔΔCt^ method.

### Protein detection by ELISA

Hippocampus tissue homogenate (10% (w/v) in 0.1 M PBS, pH 7.4) was prepared. Total proteins were measured using bicinchoninic acid (BCA) protein assay kit (Sigma-Aldrich, USA) for all samples. Quantitative measurement of the concentration of brain-derived neurotropic factor (BDNF) was conducted using a BDNF ELISA assay kit (Bioassay, Biotech, CO., Ltd Hangzhou, China). Quantitative measurement of the concentration of inflammatory markers: tumor necrosis factor-α (TNF-α) and interleukin-6 (IL-6) were conducted in hippocampus tissue homogenate by the ELISA assay kit (Bioassay, Biotech, CO., Ltd, Hangzhou, China). Moreover, hippocampal functional acetylcholine was assessed by investigating acetylcholinesterase (AChE). AChE was measured to assess the production of acetylcholine from functional neuron using AChE ELISA assay kit (Aviva System Biology, Corp, USA). All ELISA procedures were done by Hyprep Automated ELISA system (Hyperion Inc, Miami, FL) according to the manufacturer’s instructions.

### Assessment of oxidative stress

Hippocampal tissue homogenate and total protein expressions were quantified. The antioxidant status was investigated in hippocampal tissue homogenate by measuring both of reduced glutathione (GSH) level, GSH peroxidase (GPx), and super oxide dismutase (SOD) activity using commercial kits (Biodiagnostic, Cairo, Egypt) according to the manufacturer’s instructions.

### miRNA isolation from exosomes, library generation, sequencing, and analysis

miRNAs were isolated using miRNEasy kit (Qiagen, Germany) as per the manufacturer’s instruction. Pooled exosome preparations were pretreated with RNase A (Sigma-Aldrich) for 1 h to degrade unprotected RNAs. cDNA libraries for sequencing were prepared using the TruSeq Small RNA Sample Preparation Kit (Illumina, The Netherlands) following the manufacturer’s instructions. Amplified cDNA constructs were purified on 6% PAGE gel, and DNA molecules corresponding to 15–56 nucleotide transcripts were excised, eluted from gel, and concentrated by ethanol precipitation. Libraries were validated on the Bioanalyzer using the High Sensitivity DNA Chip (Agilent) and equimolarly pooled for the sequencing run. Sequencing was performed on a HiSeq 2000 (Illumina) paired end 100 cycle (PE100) run.

Analysis of sequencing results was performed as previously described [43]. Briefly, our analysis started by trimming the adapter sequences from the 3′ ends of raw data using cutadapt (v1.1) [44]. Only reads that have passed quality control were used in subsequent analyses. GENCODE v.15 [45] was used for genome annotation. miRBase (v.19) was used for annotating miRNA [46]. The most abundant miRNAs were detected after normalization of the read counts per million reads. The miRNA list was fed into the Ingenuity Pathway Analysis (IPA)® software and miRPathDB (10.1093/nar/gkz1022) to detect key pathways and target genes associated with our discovered miRNA expressed in exosomes and identify the target genes, pathways and diseases associated with these particular miRNAs.

### Statistical analysis

Parametric data were expressed as mean ± SEM. Homogeneity Shapiro-Wilk test was used to test the normal distribution of data. Comparison of parametric data was done between more than two groups by analysis of variance (ANOVA) using post hoc test (Tukey’s multiple comparison test) to compare individual groups. Non-parametric data was presented as medians and interquartile range and analyzed by Kruskal–Wallis test followed by Dunn’s post hoc test. Statistical analyses were implemented using GraphPad software (Instat, 3.06 version) and the IBM SPSS statistics (V.19.0, IBM Corp., USA, 2010). A probability of *p* value < 0.05 was considered to be statistically significant.

## Results

### Characterization of isolated BMSCs and BMSCs-Exo and localization of BMSCs in the brain

**Fig. 2:**
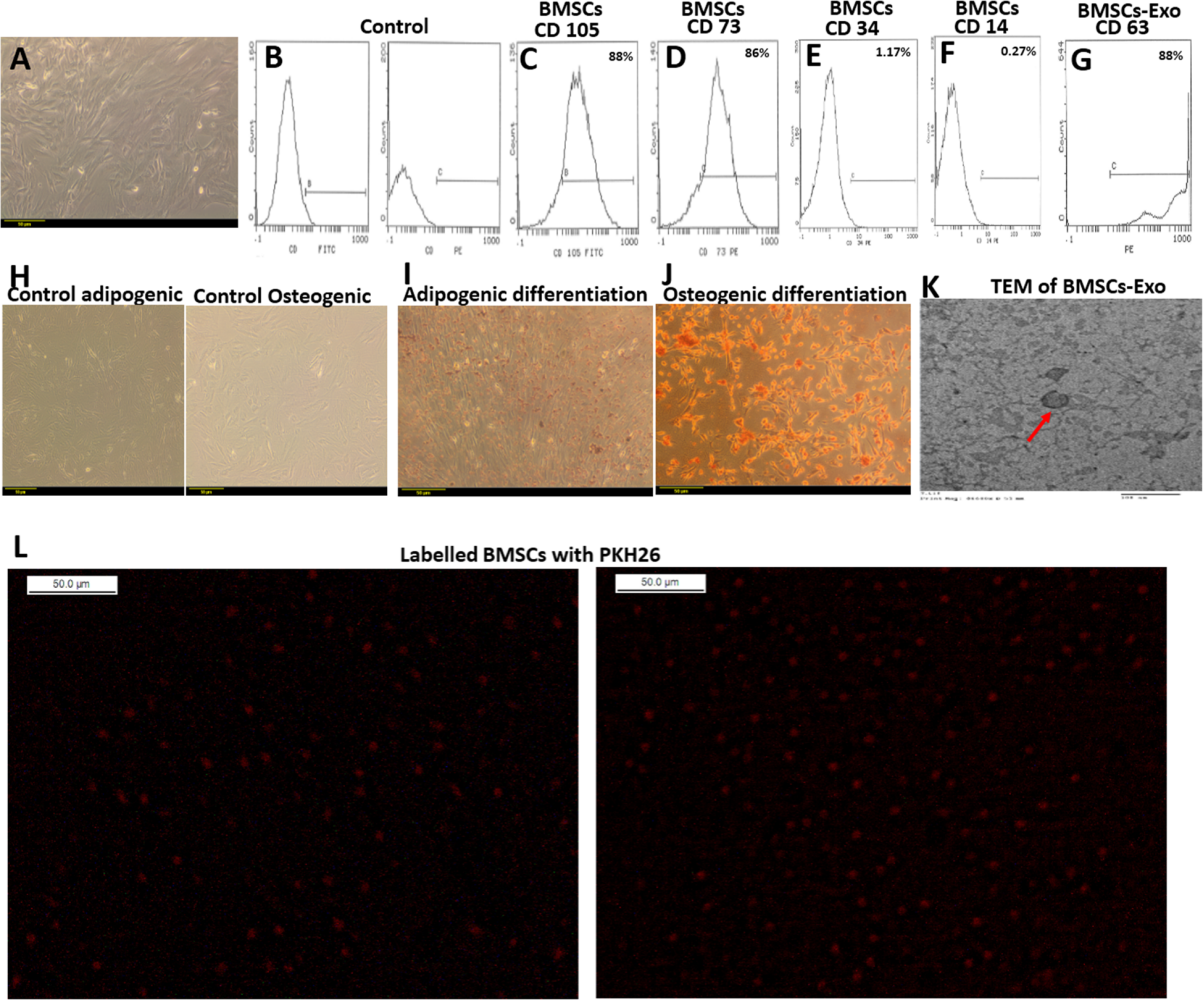
Characterization, differentiation, and migration of bone marrow stem cells (BMSCs). **A** A representative image showing fibroblastic morphology of BMSCs (× 200). **B** Flow cytometry curve showing a control, left side: gating of unstained cells at FITC, right side: gating of unstained cells at PE. **C** Flow cytometry curve showing the expression of CD105 in BMSCs. **D** Flow cytometry curve showing the expression of CD73 in BMSCs. **E** Flow cytometry curve showing the expression of CD34 in BMSCs. **F** Flow cytometry curve showing the expression of CD14 expression in BMSCs. **G** Flow cytometry curve showing the expression of CD63 in exosome produced by BMSCs. **H** Control BMSCs cultured in LDMEM supplemented with 10% bovine serum, 100 U/mL penicillin, and 100 μg/mL streptomycin, left side: stained with Oil Red and right side: stained with Alizarin Red (× 200). **I** BMSCs stained with Oil Red showing adipogenic differentiation of BMSCs (× 200). **J** BMSCs stained with Alizarin Red showing osteogenic differentiation of BMSCs (× 200). **K** Transmission electron microscope image of exosome produced by BMSCs showing vesicular structure (arrow). **L** Localization of PKH26-labeled BMSCs in the hippocampal regions of the brain tissues of rats (× 200) (*n* = 4)

BMSCs were isolated from rat’s bone marrow as detailed in the “Materials and methods” section. The isolated and cultured BMSCs were successfully identified by their ability to proliferate in culture with an attached well-spread fibroblastic morphology as shown in Fig. 2A. Additionally, BMSCs were negative for the hematopoietic markers CD34 and CD14 while being positive for mesenchymal markers CD105 and CD73 (Fig. 2B–F). The expression of the exosomal marker protein, CD63, was also confirmed in BMSCs-Exo as revealed in Fig. 2G. Adipogenic and osteogenic differentiations were successfully attained as shown by Oil Red and Alizarin Red staining in Fig. 2I,J, as compared to undifferentiated BMSCs cultured in normal growth (Fig. 2H). In addition, exosomes secreted by BMSCs were successfully isolated and characterized by electron microscopy where they showed vesicular structures (Fig. 2K).

Since our aim is to assess the therapeutic potential of BMSCs and BMSCs-Exo in chemobrain, this study induced chemobrain in rats using DOX followed by injections with either BMSCs or BMSCs-Exo. First, the localization of BMSCs into the brain was assessed. Brain tissue was examined under a florescence microscope to detect fluorescent-labeled BMSCs. Two sections per each rat were investigated, and labeled BMSCs were observed in the hippocampal brain regions (Fig. 2L). This finding is mainly due to the fact that this area is more sensitive to injury and neuronal pyknosis.

### BMSCs and BMSCs-Exo restored long-term and short-term spatial memory functions in DOX-induced chemobrain

**Fig. 3:**
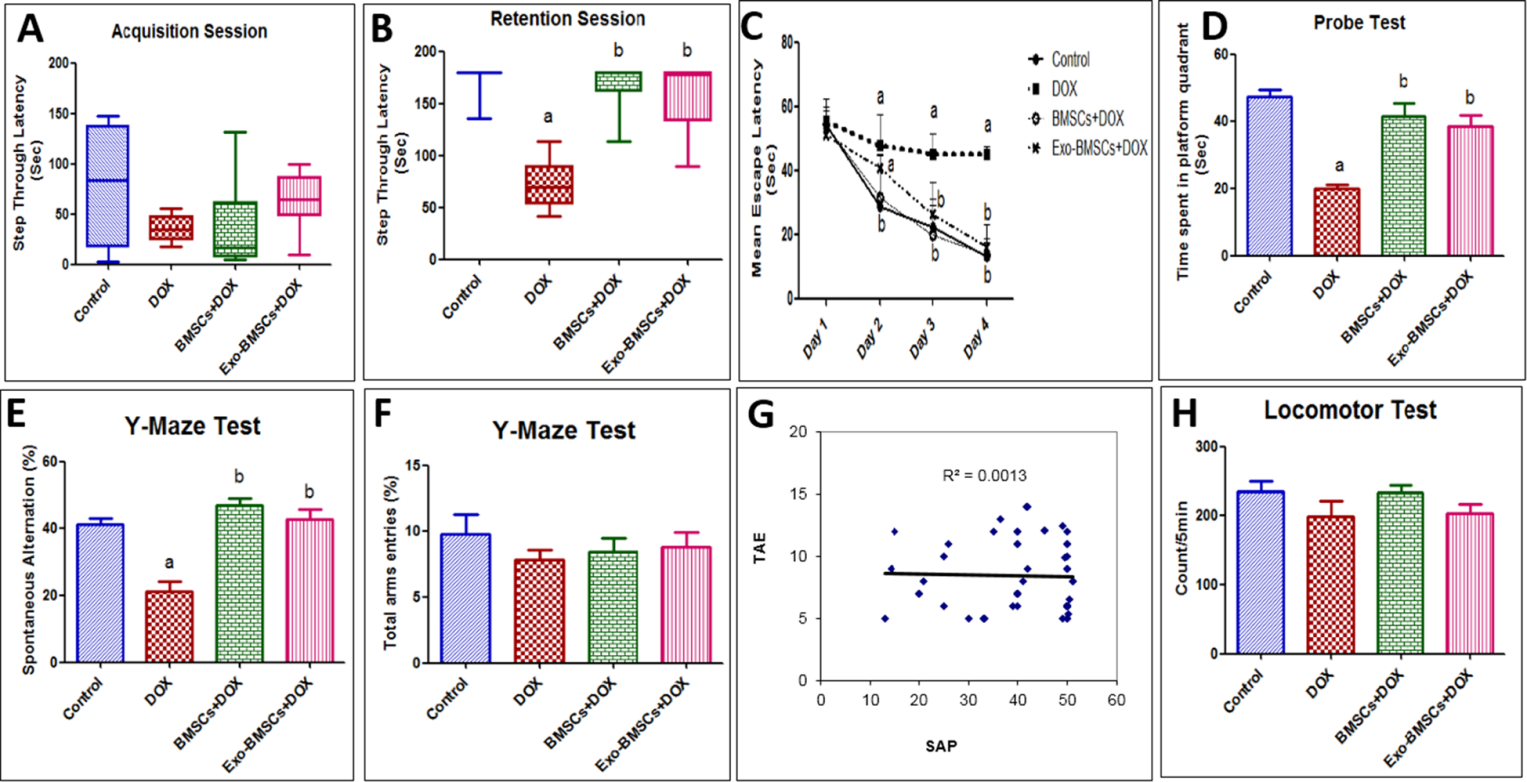
Bone marrow stem cell (BMSC) and their exosome (BMSCs-Exo) treatment reverse behavioral changes induced by doxorubicin (DOX) in rats. **A**, **B** Box plots representing results from step-through passive avoidance behavioral test. **C** A scatter plot representing results from Morris water maze test. **D** A bar chart representing results from the probe test. **E** A bar chart representing Y-maze % of alternation (SAP). **F** A bar chart representing Y-maze total arm entries (TAE). **G** A scatter plot representing the correlation between SAP and TAE and **H** a bar chart representing results from the locomotor activity test. For **A** and **B**, data are presented as medians (25th, 75th percentile) and statistical analysis was carried out using Kruskal–Wallis non-parametric test followed by Dunn’s test (*n* = 8). For **C**–**H,** data are presented as mean ± SEM (*n* = 8). For **C**, statistical analysis was performed using repeated two-way ANOVA followed by the Bonferroni post-tests as post hoc test. For **D–F** and **H**, statistical analysis was performed using one-way ANOVA followed by Tukey’s test as post hoc test (*n* = 8). ^a^ Significantly different from the control group at *p* < 0.05. ^b^ Significantly different from DOX group at *p* < 0.05. For **G**, statistical analysis was performed using Pearson’s correlation

Next, we assessed learning and memory impairments induced by DOX by PA task which assesses the cognitive functions. On the training session, there was no statistically significant difference in the step-through latency among different treated groups as shown in Fig. 3A. However, during the test session, DOX-induced chemobrain group showed significant shortening in the step-through latency (Fig. 3B). Interestingly, BMSC- or BMSCs-Exo-treated groups significantly restored the cognitive function indicated by a significant increase in the step-through latency (Fig. 3B). Moreover, there was no significant difference between the latency in BMSCs and BMSCs-Exo groups which indicates that the beneficial action of BMSCs is exerted through their secreted exosomes.

Hippocampal-dependent learning, including acquisition of spatial memory and long-term spatial memory, was significantly reduced by DOX administration as compared to control group as shown in MWM test (Fig. 3C). Whereas a significant increase in the mean escape latency was observed in the second, third, and fourth days as compared to the control group (Fig. 3C). BMSC treatment restored long-term spatial memory which is reflected by decreasing mean escape latency in the second, third, and fourth days when compared to DOX-induce chemobrain. Likewise, BMSCs-Exo restored long-term spatial in the third and fourth days when compared to DOX-induced chemobrain (Fig. 3C).

Besides, BMSCs significantly improved spatial memory functions as reflected by the probe test which showed a significant decrease in the time spent in the platform quadrant in the DOX group as compared to the control group (Fig. 3D). Both BMSC- and BMSCs-Exo-treated groups significantly increased the time spent in the platform quadrant as compared to the DOX-induced chemobrain group (Fig. 3D).

Short-term spatial memory functions were studied by investigating the SAP in Y-Maze test in different groups. It was found that DOX-induced chemobrain showed a significant decrease in SAP as compared to the control group (Fig. 3E). On the other hand, treatment with either BMSCs or BMSCs-Exo restored short-term memory function as showed by a significant increase in SAP as compared to the DOX-induced chemobrain group (Fig. 3E). There were no significant differences between the BMSCs or BMSCs-Exo, further confirming that the therapeutic effects of BMSCs might be exerted mainly through their exosomes. Furthermore, no significant differences were found among groups in Y-Maze TAE between all the studied groups (Fig. 3F). Additionally, no correlation was found between SAP and TAE between all studied groups reflecting that any differences in spontaneous locomotor activity did not impact quantification of spontaneous alternation (Fig. 3G).

Concerning locomotion activity, our results found that it is not affected by DOX-induced chemobrain as confirmed by previous studies [23]. In alignment, this study showed no statistically significant difference between DOX-induced chemobrain, the control group and BMSC- and BMSCs-Exo-treated group on locomotor activity in rats (Fig. 3H).

### BMSCs and BMSCs-Exo mitigated histopathological alteration in DOX-induced chemobrain

**Fig. 4:**
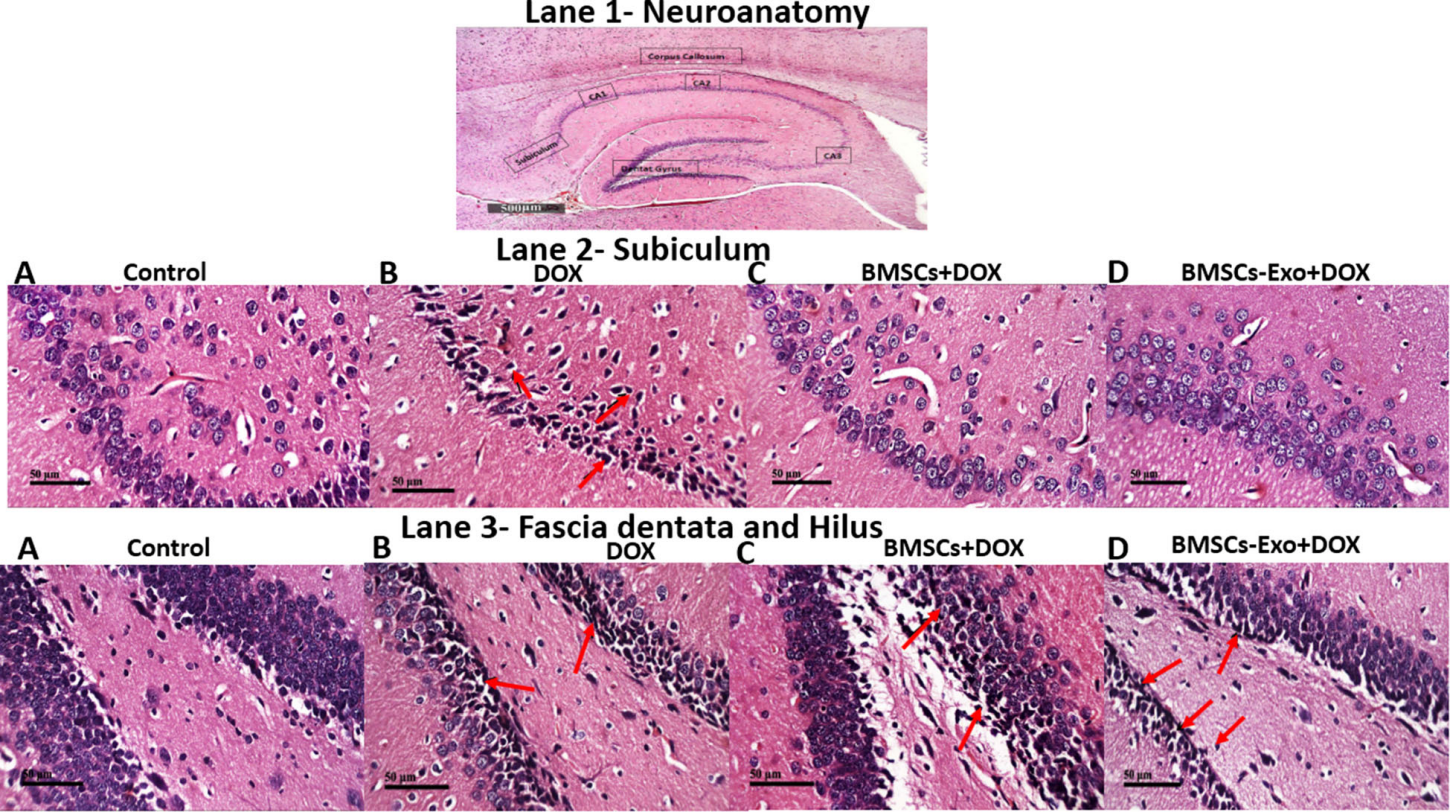
Histopathological analysis of rat brain sections using H&E staining. Lane (1) (× 100): Neuroanatomy of brain areas being investigated to show brain structures in the representative micrographs. Lane (2) (× 400): **A** Control group showed normal histological structure of the neurons in the subiculum. **B** DOX-induced chemobrain group showed nuclear pyknosis and degeneration observed in diffuse manner all over the neurons in the subiculum (arrow). **C** BMSC-treated DOX group showed normal subiculum histological features. **D** BMSCs-Exo treated DOX group showed no histological alterations in the subiculum regions. Lane (3) (× 400): **A** the control group showed normal histological structure of the neurons in the fascia dentata and hilus of the hippocampus. **B** DOX-induced chemobrain group showed nuclear pyknosis and degeneration observed in fascia dentata and hilus (arrow) of the hippocampus. **C** BMSC-treated DOX group showed few nuclear pyknosis and degeneration in fascia dentata and hilus of the hippocampus (arrow). **D** BMSCs-Exo-treated DOX group showed nuclear pyknosis and degeneration was shown in some neurons in fascia dentata and hilus of the hippocampal region (arrow) (*n* = 4)

Histopathological examination of brain sections showed that systemic DOX administration resulted in nuclear pyknosis and degeneration observed in a diffuse manner all over the neurons in the subiculum, fascia dentata, and hilus of the hippocampus (Fig. 4). In contrast, both BMSC- and BMSCs-Exo-treated groups showed no histopathological alteration in subiculum regions (Fig. 4). However, some nuclear pyknosis and degeneration were shown in some neurons in fascia dentata and hilus of the hippocampal regions in the BMSCs-Exo-treated group (Fig. 4).

**Fig. 5:**
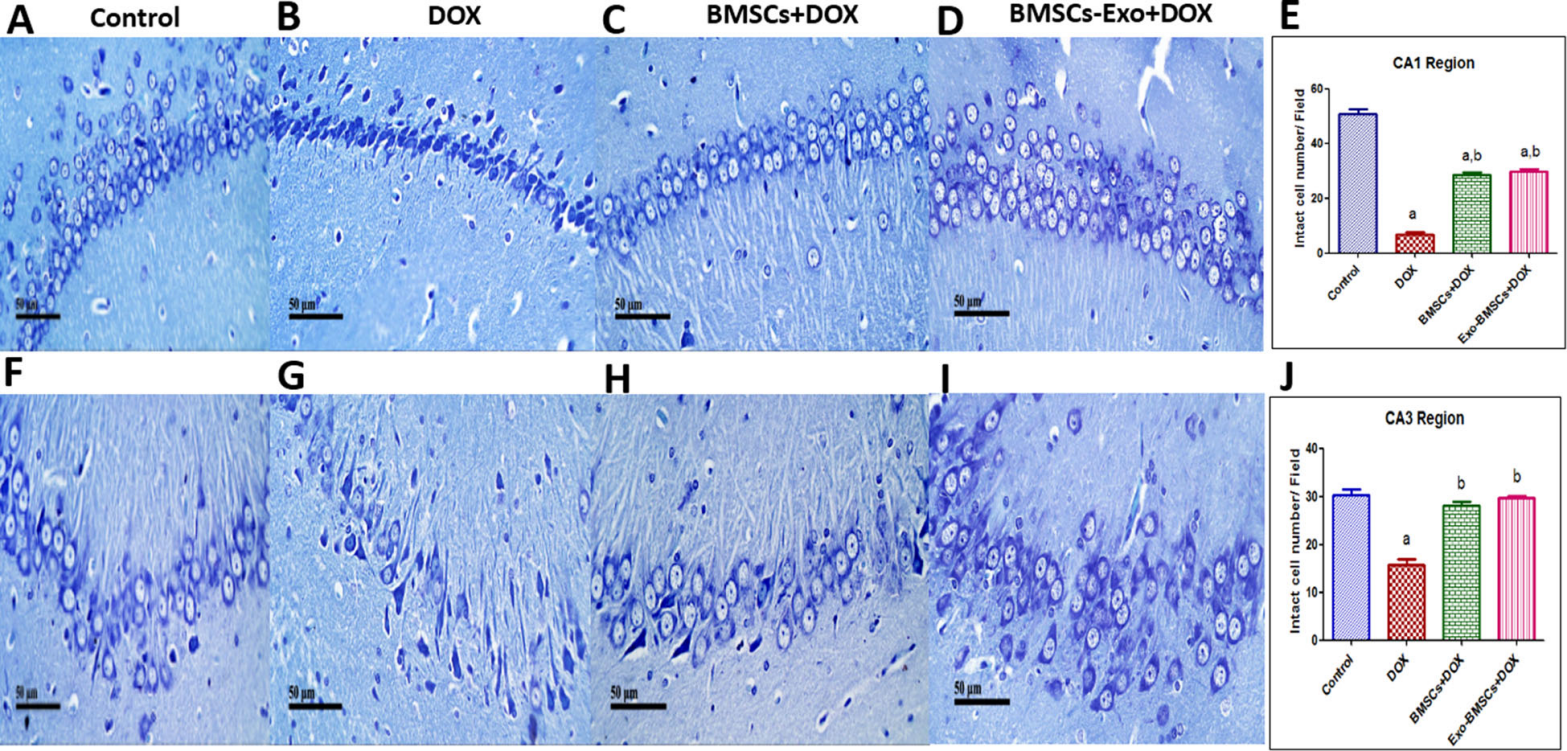
Bone marrow stem cell (BMSC) and their exosome (BMSCs-Exo) treatment reverse neural damage induced by doxorubicin (DOX) in rats. Representative images of toluidine blue-stained brain tissue sections (× 400) of **A** the control group showing normal histological structure showing intact neurons in CA1 region, **B** DOX-induced chemobrain group showing decrease in intact neuron in CA1 region, **C** BMSC treatment group showing significant restoration in intact neurons in CA1 region, and **D** BMSCs-Exo treatment group showing significant restoration in intact neurons in CA1 region. **E** A bar chart reflecting the quantitative image analysis for intact neurons in CA1 region expressed intact cell number per field. Representative images of toluidine blue-stained brain tissue sections (× 400) of **F** control group showing normal histological structure showing intact neurons in CA3 region, **G** DOX-induced chemobrain group showing decrease in intact neuron in CA3 region, **H** BMSC treatment group showing significant restoration in intact neurons in CA3 region, and **I** BMSCs-Exo treatment group showing significant restoration in intact neurons in CA3 region. **J** A bar chart reflecting the quantitative image analysis for intact neurons in CA3 region expressed intact cell number per field. For **E** and **J**, statistical analysis was performed using one-way ANOVA followed by Tukey’s test as post hoc test (*n* = 4). Data are presented as mean ± SEM. ^a^ Significantly different from the control group at *p* < 0.05. ^b^ Significantly different from DOX group at *p* < 0.05

Assertively, a significant increase in neural degeneration and neural pyknosis in the DOX-induced chemobrain group was recorded in CA1 area (Fig. 5B,E) and CA3 area (Fig. 5G,J) as compared to the control group. In contrast, the number of intact neurons was increased in CA1 area (Fig. 5C,E) and in CA3 area (Fig. 5H,J) in the BMSC-treated group when compared to the DOX-induced chemobrain group.

Similarly, the BMSCs-Exo-treated group showed a significant increase in the number of intact neurons in CA1 area (Fig. 5D,E) and in CA3 area (Fig. 5I,J) when compared to the DOX-induced chemobrain group.

### BMSCs and BMSCs-Exo reversed DOX-induced neural demyelination

**Fig. 6:**
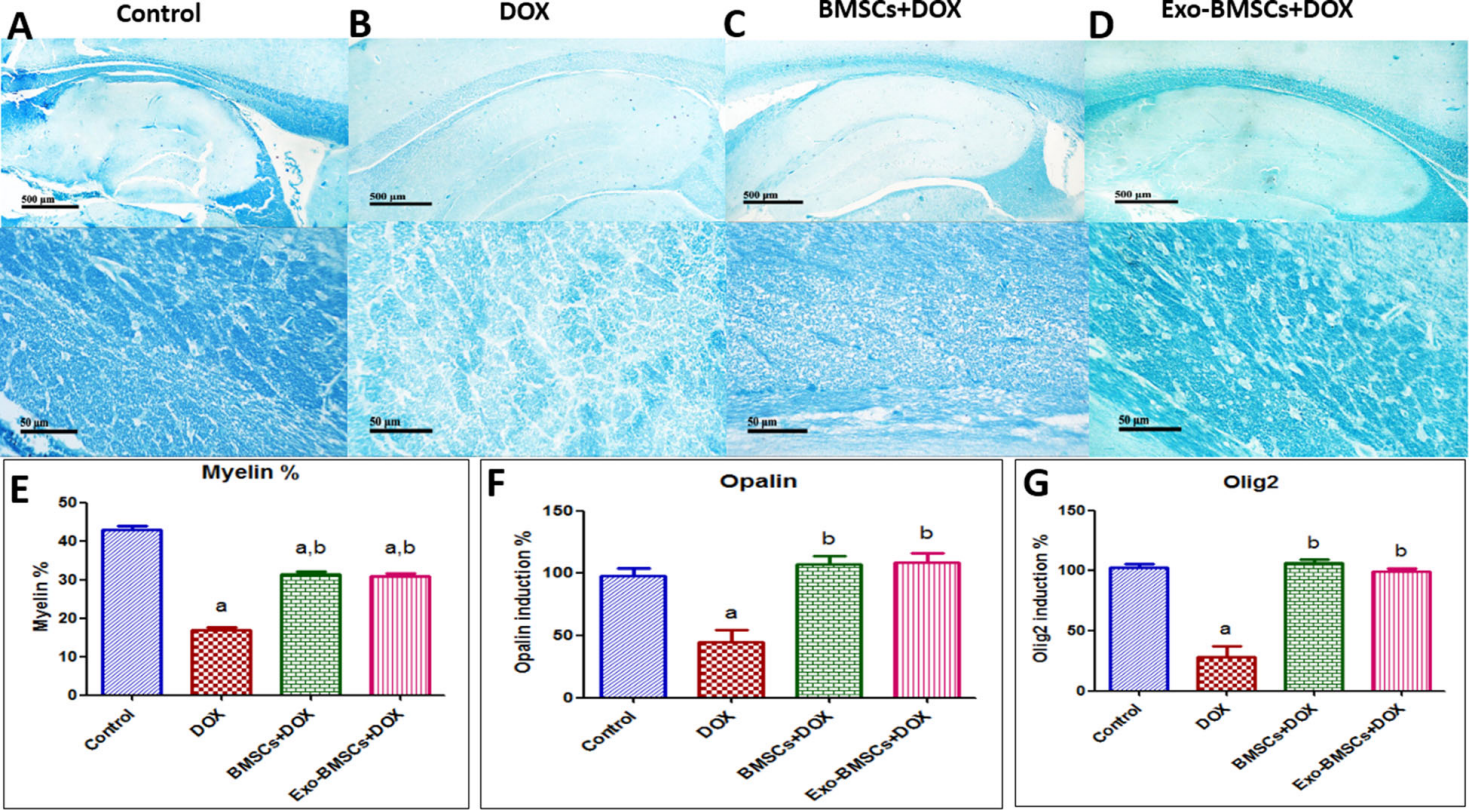
Bone marrow stem cell (BMSC) and their exosome (BMSCs-Exo) treatment prevent neural demyelination induced by doxorubicin (DOX) in rats. Representative images of Luxol fast blue-stained sections (× 100 and × 400) of **A** the control group showing positive stained myelinated nerve fibers in the corpus callosum region, **B** DOX-induced chemobrain group showing decrease in positively stained myelinated nerve fibers in the corpus callosum region, **C** BMSC treatment group showing restoration of positively stained myelinated nerve fibers in the corpus callosum region, and **D** BMSCs-Exo treatment group showing restoration of positively stained myelinated nerve fibers in the corpus callosum region. **E** A bar chart reflecting the quantitative image analysis for positive stained myelinated nerve fibers in the corpus callosum region expressed as area percent. **F** A bar chart representing the % of mRNA expression induction of Opalin in different rat groups. **G** A bar chart representing the % of mRNA expression induction of Olig-2 in different rat groups. For **E**, **F**, and **G**, statistical analysis was performed using one-way ANOVA followed by Tukey’s test as post hoc test (*n* = 6). Data are presented as mean ± SEM. ^a^ Significantly different from the control group at *p* < 0.05. ^b^ Significantly different from the DOX group at *p* < 0.05

Neural myelination was examined in the corpus callosum regions using Luxol fast blue-stained sections. Neural myelination was significantly decreased in the DOX-induced chemobrain when compared to the control group (Fig. 6B,E) while both the BMSC- and the BMSCs-Exo-treated groups significantly regenerated neural myelination as compared to the DOX-induced chemobrain group (Fig. 6A–E).

Furthermore, we assessed the expression of neural myelin genes and found a significant decrease in Opalin gene expression in the chemobrain group as compared to the control group (Fig. 6F), while a significant increase was found in the BMSC- and in the BMSCs-Exo-treated groups as compared to DOX-induced chemobrain group (Fig. 6F). Furthermore, the oligodendrocyte differentiation marker, Olig-2, showed a significant decrease in the DOX-induced chemobrain group as compared to the control group while it showed a significant increase in the BMSC- and in the BMSCs-Exo-treated groups as compared to the DOX-induced chemobrain group (Fig. 6G).

### BMSCs and BMSCs-Exo reverted DOX-induced hippocampal neurotropic growth factor depletion

**Fig. 7:**
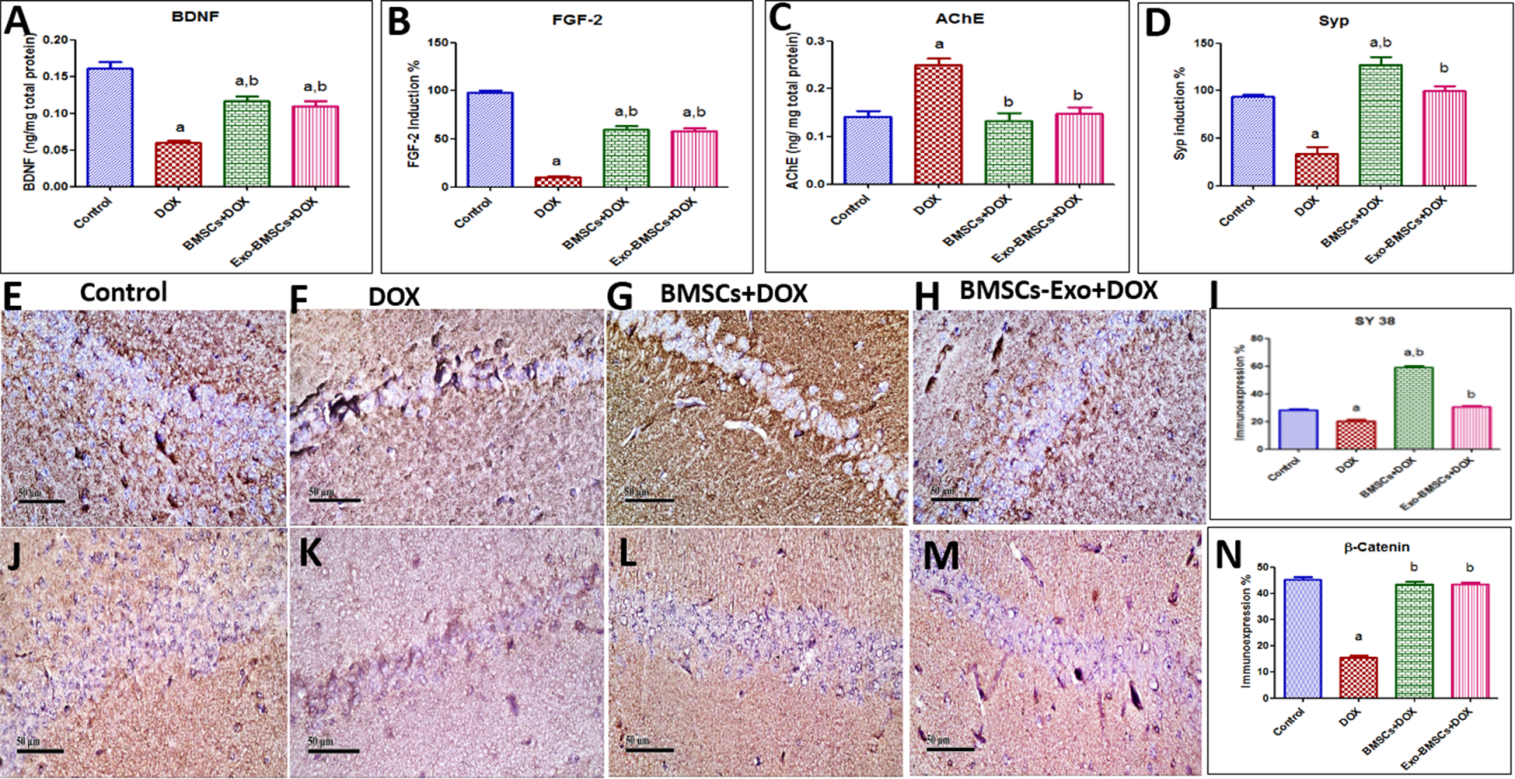
Bone marrow stem cell (BMSC) and their exosome (BMSCs-Exo) treatment restore hippocampal neurotropic and synaptic growth factor, functional neurotransmitters, and hippocampal β-catenin expression in doxorubicin (DOX)-induced chemobrain in rats. **A** A bar chart representing the protein expression induction of BDNF in different rat groups. **B** A bar chart representing the % of mRNA expression induction of FGF-2 in different rat groups. **C** A bar chart representing the quantification of acetylcholinesterase (AChE) in different rat groups. **D** A bar chart representing the % of mRNA expression induction of Syp in different rat groups. Representative image reflecting immunohistochemical staining of SY 38 in **E** control group showing moderate expression of SY 38 in hippocampal sections, **F** DOX-induced chemobrain group showing minimal expression of SY 38, **G** group treated with BMSCs showing marked expression of SY 38, and **H** group treated with BMSCs-Exo showing moderate expression of SY 38. **I** A bar plot reflecting the quantitative image analysis for SY 38 immunohistochemical staining expressed as area percent. Representative image reflecting immunohistochemical staining of β-catenin in **J** the control group showing marked expression of β-catenin in hippocampal region, **K** DOX-induced chemobrain group showing minimal expression of β-catenin in hippocampal region, **L** BMSC-treated group showing marked expression of β-catenin, and **M** BMSCs-Exo-treated group showing marked expression of β-catenin. **N** A bar plot reflecting quantitative image analysis for β-catenin immunohistochemical staining expressed as area percent. Data in **A**–**D** (*n* = 6), **I**, **N** (*n* = 4) are presented as mean ± SEM. Statistical analysis was performed using one-way ANOVA followed by Tukey’s post hoc test. ^a^ Significantly different from the control group at *p* < 0.05. ^b^ Significantly different from the DOX group at *p* < 0.05

Hippocampal neurotropic growth factor as well as synaptic markers was significantly regenerated by BMSCs and BMSCs-Exo administration, whereas BDNF was significantly depleted in the DOX-induced chemobrain group compared to the control group. However, BMSCs and BMSC-Exo successfully increased BDNF gene expression (Fig. 7A).

In the same line, FGF-2 gene expression was significantly decreased in the DOX-induced chemobrain group as compared to the control group and was restored upon BMSCs or BMSCs-Exo treatment (Fig. 7B).

### BMSCs and BMSCs-Exo increased functional neurotransmitters levels

AChE is an enzyme that metabolizes Ach and prevents it from exerting its actions, whereas ACh is essential for neuro-communications and cognition. The inhibitory effect of AChE was shown to be significantly increased in the DOX-induced chemobrain as compared to the control group which reflects a reduction of the ACh neurotransmitter (Fig. 7C). A significant decrease in AChE levels is reported in the BMSC- or the BMSCs-Exo-treated group as compared to the DOX-induced chemobrain group (Fig. 7C).

### BMSCs and BMSCs-Exo upregulated hippocampal synaptic growth factors depletion in DOX-induced chemobrain

Gene expression of Syp was significantly decreased in the DOX-induced chemobrain group as compared to the control group while BMSCs and BMSCs-Exo successfully increased Syp gene expression as compared to the DOX-induced chemobrain group (Fig. 7D).

In the same line, immune expression of the presynaptic protein synaptophysin (SY 38) was determined as well. Our results show a significant decrease of SY 38 expression in DOX-induced chemobrain as compared to the control group while a significant increase in SY 38 expression was detected in BMSC- and BMSCs-Exo-treated groups compared to the DOX-induced chemobrain group (Fig. 7E–I).

### BMSCs and BMSCs-Exo abrogated DOX-induced neurodegeneration via multiple signaling crosstalk orchestrated by its miRNA cargo

**Fig. 8:**
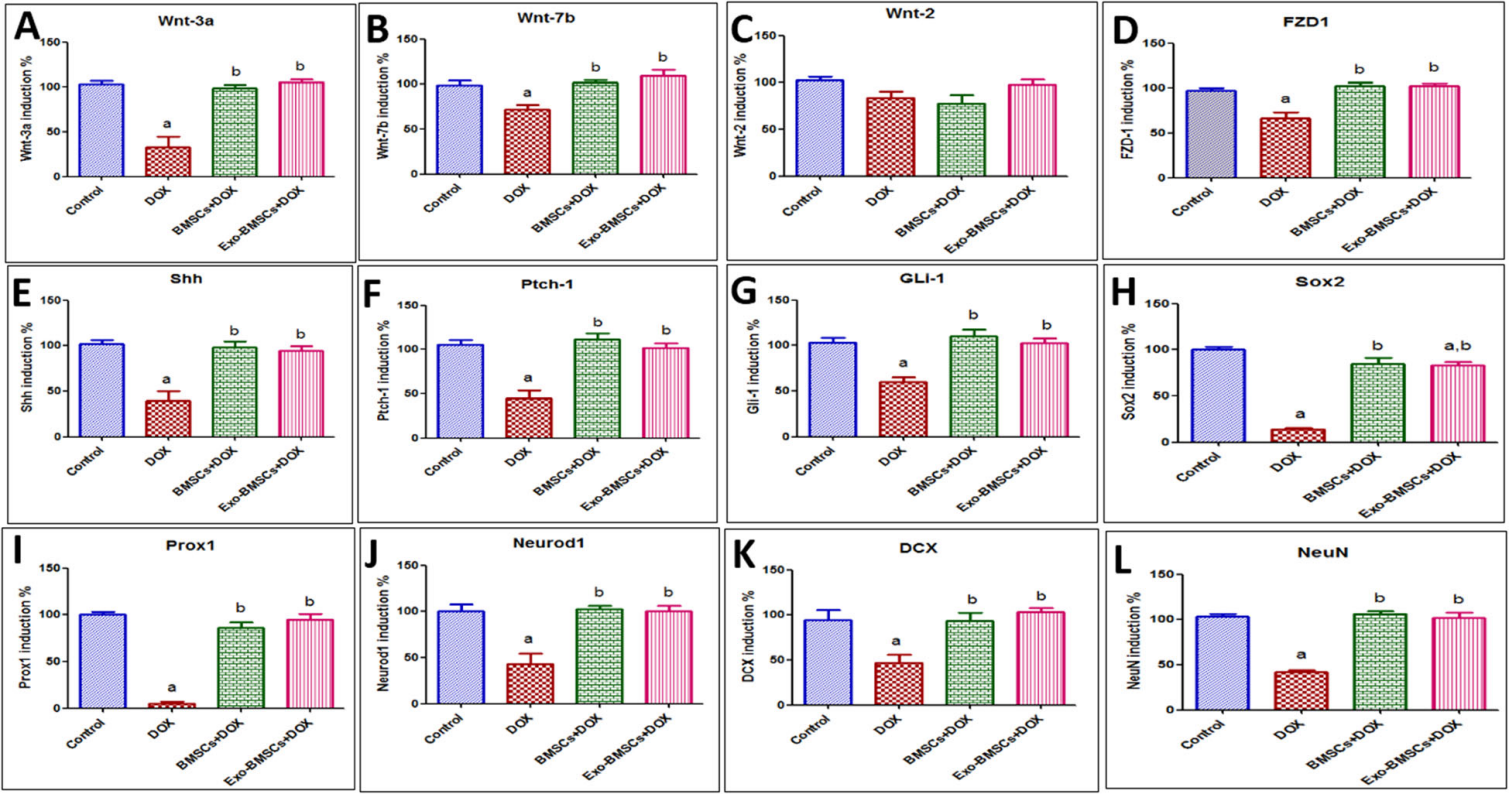
Effects of bone marrow stem cell (BMSC) and their exosome (BMSCs-Exo) treatment on Wnt/β-catenin and hedgehog signaling genes and transcription factors in doxorubicin (DOX)-induced chemobrain in rats. **A** A bar chart representing the % of mRNA expression induction of Wnt-3a in different rat groups. **B** A bar chart representing the % of mRNA expression induction of Wnt-7b in different rat groups. **C** A bar chart representing the % of mRNA expression induction of Wnt-2 in different rat groups. **D** A bar chart representing the % of mRNA expression induction of FZD1 in different rat groups. **E** A bar chart representing the % of mRNA expression induction of sonic hedgehog (shh) in different rat groups. **F** A bar chart representing the % of mRNA expression induction of patched-1 (Ptch-1) in different rat groups. **G** A bar chart representing the % of mRNA expression induction of the transcription factor Gli-1 in different rat groups. **H** A bar chart representing the % of mRNA expression induction of Sox2 in different rat groups. **I** A bar chart representing the % of mRNA expression induction of Prox1 in different rat groups. **J** A bar chart representing the % of mRNA expression induction of NeuroD1 in different rat groups. **K** A bar chart representing the % of mRNA expression induction of DCX in different rat groups. **L** A bar chart representing the % of mRNA expression induction of NeuN in different rat groups. Data are presented as mean ± SEM. Statistical analysis was performed using one-way ANOVA followed by Tukey’s post hoc test (*n* = 6). ^a^ Significantly different from the control group at *p* < 0.05. ^b^ Significantly different from the DOX group at *p* < 0.05

In our effort to determine the underlying pathways that are responsible for neurodegeneration in chemobrain, we investigated mechanisms related to the Wnt/ β-catenin and hedgehog pathways. Starting with the Wnt/ β-catenin pathway, β-catenin expression was explored by immunohistochemistry and showed a significant decrease in DOX-induced chemobrain as compared to the control group (Fig. 7J–N). Meanwhile, a significant increase in the immune expression of β-catenin was observed in the BMSC- and in the BMSCs-Exo-treated groups as compared to the DOX-induced chemobrain group. Besides, a significant decrease was found in Wnt3a, Wnt7b, and FZD1 gene expression in DOX-induced chemobrain as compared to the control group (Fig. 8A,B,D). These changes were reversed upon treatment with BMSCs or BMSC-Exo. However, no significant changes were observed in Wnt2 gene expression between our studied groups (Fig. 8C).

Concerning the hedgehog pathway, hedgehog signaling was shown to be significantly decreased in DOX-induced chemobrain evidenced by decreased Shh expression as compared to the control group. Besides, significant reductions in Ptch1 receptor gene expression and the transcription factor Gli-1 were observed in the DOX-induced chemobrain group as compared to the control group (Fig. 8E–G). Noteworthy, these changes were also reversed upon BMSC or BMSCs-Exo treatment.

Regarding crucial transcription factors in neural development, our results show reduced expression of Sox2, Prox1, and Neurod1 in DOX-induced chemobrain as compared to the control group (Fig. 8H–J). The neuronal migration marker, DCX, which is expressed by neuronal precursor cells in immature neurons was significantly decreased in DOX-induced chemobrain as compared to the control group (Fig. 8K) while mature neurons expressing NeuN showed a significant decrease in DOX-induced chemobrain as compared to the control group (Fig. 8L). Reversibly, BMSC or BMSCs-Exo treatment showed a significant increase in Sox2, Prox1, and Neurod1 gene expression as compared to DOX-induced chemobrain (Fig. 8H–J). Moreover, DCX gene expression was significantly increased in the treated groups as compared to DOX-induced chemobrain (Fig. 8K). Finally, the maturation neural marker, NeuN, shows a significant increase in gene expression in the BMSC- and the BMSCs-Exo-treated groups as compared to DOX-induced chemobrain (Fig. 8L).

**Fig. 9:**
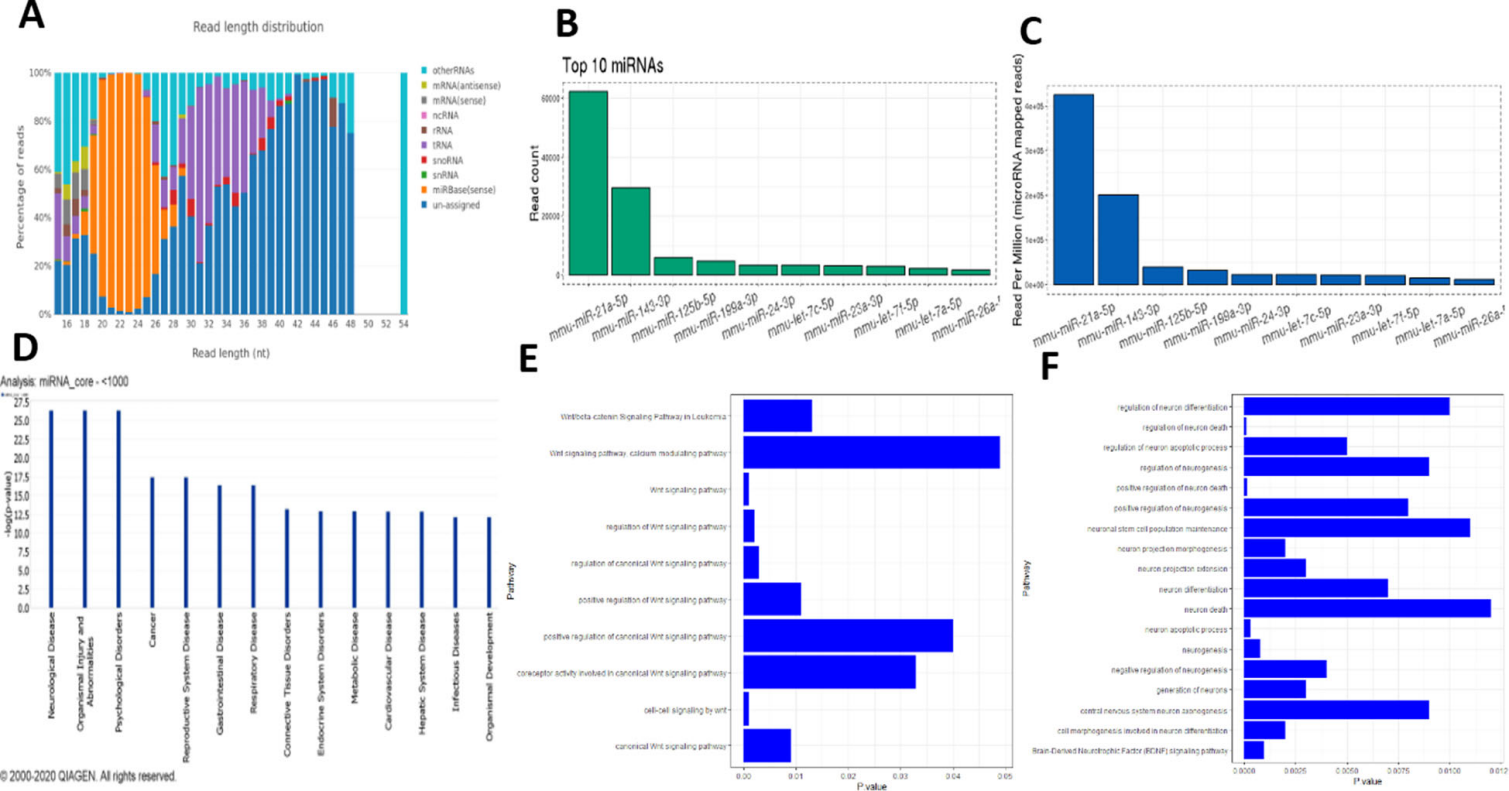
Analysis of the miRNA cargo of exosomes secreted by bone marrow stem cells (BMSC-Exo). **A** A bar plot representing the distribution of sizes of the sequenced RNA molecules in BMSC-Exo. **B** A bar plot reflecting the counts of the top 10 most abundant miRNA in BMSC-Exo. **C** A bar plot reflecting the normalized counts of the top 10 most abundant miRNA in BMSC-Exo. **D** A bar plot reflecting the association significance between the discovered miRNA and disease as predicted by IPA analysis. **E** Association between miR-21-5p and Wnt/β-catenin signaling based on interaction data from the miRPathDB (10.1093/nar/gkz1022). **F** Association between miR-21-5p and neural hallmarks based on interaction data from the miRPathDB (10.1093/nar/gkz1022)

Next, we aimed at discovering the mechanistic pathways responsible for the BMSCs and BMSCs-Exo beneficial actions. Next-generation sequencing (NGS) was performed to identify the miRNA content of the BMSCs-Exo. Our results show that exosomes are enriched in miRNA with the majority ranging in length between19 and 26 nucleotides (Fig. 9A). The top 10 most abundant miRNA counts and normalized counts are illustrated in Fig. 9B,C. Next, we performed IPA analysis of the most abundant miRNAs (normalized count > 1000). IPA analysis of the most abundant miRNA expressed in the exosomes showed that 17 of the 18 highly expressed miRNAs are most significantly associated with neurological and psychological disorders (Fig. 9D).

Besides, a network reflecting the connection between the discovered miRNA in BMSC-Exo and sporadic amyotrophic lateral sclerosis, amyotrophic lateral sclerosis, and progressive motor neuropathy were illustrated (Suppl. Fig. 1). Moreover, a table of the disease categories associated with the most abundant miRNA in BMSC exosomes is shown in Suppl. Fig. 1.

Interestingly, miR-21-5p, which is the most abundant miRNA expressed in BMSCs-Exo, mediates its function through regulation of the Wnt/β-catenin signaling pathway as discovered by our IPA analysis and based on interaction data from the miRPathDB (10.1093/nar/gkz1022) (Fig. 9E) and as described in literature [47]. Besides, miR-21-5p is found to be significantly associated with neural hallmarks as well as BDNF signaling pathway based on interaction data from the miRPathDB (10.1093/nar/gkz1022) (Fig. 9F). miR-21-5p gene targets (Suppl. Table. 1) and the pathways affected by miR-21-5p are provided in supplementary files (Suppl. Table. 2).

miR-125b-5p, miR-199a-3p, miR-24-3p, and let-7a-5p, enriched in the BMSCs-Exo, were found to mediate their effect through regulation of the Wnt/β-catenin signaling pathway. Besides, miR-30c-5p, miR-29b-3p, miR-16-5p, miR-221-3p, miR-181a-5p, miR-145-5p, miR-31-5p, and miR-34a-5p were also found to a lesser extent in the exosomes and were reported to mediate their effect through Wnt/β-catenin signaling pathway based on interaction data from the miRPathDB (10.1093/nar/gkz1022). In the same line, miR-125b-5p, miR-24-3p, and miR-199a-3p were found to mediate their effect through regulation of sonic hedgehog signaling pathway (Suppl. Table. 2), whereas miR-125b-5p mediates its function of sonic hedgehog signaling regulations through (Smo) (Suppl. Table. 2).

### BMSCs and BMSCs-Exo halted astrocyte activation and microglia activation

**Fig. 10:**
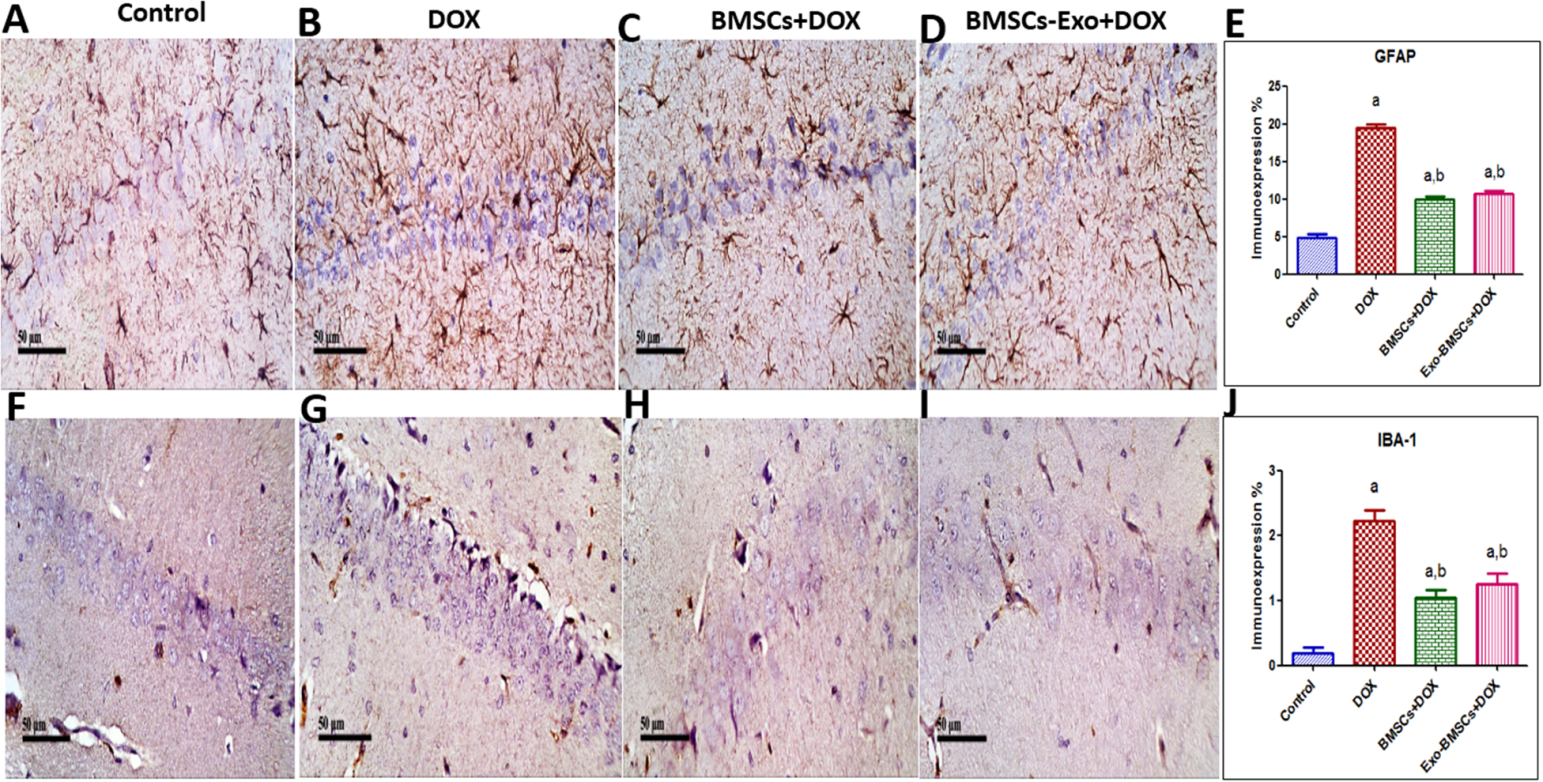
Bone marrow stem cells (BMSCs) and their exosomes (BMSCs-Exo) halt astrocytes and microglia activation in doxorubicin (DOX)-induced chemobrain in rats. Representative image reflecting immunohistochemical staining of GFAP “a marker of astrocyte activation” in **A** the control group showing minimal expression of GFAP in hippocampal region, **B** DOX-induced chemobrain group showing high expression of GFAP in hippocampal region, **C** BMSC-treated group showing moderate expression of GFAP, and **D** BMSCs-Exo-treated group showing moderate expression of GFAP. **E** A bar plot reflecting quantitative image analysis for GFAP immunohistochemical staining expressed as area percent. Representative image reflecting immunohistochemical staining of IBA-1 “a marker of microglia activation” in **F** the control group showing minimal expression of IBA-1 in the hippocampal region, **G** DOX-induced chemobrain group showing high expression of IBA-1 in hippocampal region, **H** BMSC-treated group showing moderate expression of IBA-1, and **I** BMSCs-Exo-treated group showing moderate expression of IBA-1. **J** A bar plot reflecting quantitative image analysis for IBA-1 immunohistochemical staining expressed as area percent. Data are presented as mean ± SEM (*n* = 4). Statistical analysis was performed using one-way ANOVA followed by Tukey’s test as post hoc test. ^a^ Significantly different from the control group at *p* < 0.05. ^b^ Significantly different from the DOX group at *p* < 0.05

Astrocyte activation is widely associated with reduced cognitive impairments in multiple cognitive impairment diseases [48, 49]. In that spirit, the expression of GFAP, a marker of astrocyte activation, was significantly increased in DOX-induced chemobrain group when compared to the control group (Fig. 10A–E). Conversely, BMSCs and BMSCs-Exo successfully decreased GFAP expression when compared to DOX-induced chemobrain.

Sustaining learning and memory behaviors are extensively affected by microglia activation [50]. IBA-1 was shown to be the key player in actin-crosslinking involved in membrane ruffling of microglia. Therefore, IBA-1 is considered a crucial marker for microglial activation [51]. In our study, we show defected learning by increased hippocampal expression of microglial activation marker IBA-1 in the DOX-induced chemobrain group as compared to the control group (Fig. 10F–J). Interestingly, BMSCs and BMSCs-Exo significantly decreased the expression of IBA-1 compared to the DOX-induced chemobrain group.

### BMSCs and BMSCs-Exo protected against inflammatory and apoptotic activation in the brain

**Fig. 11:**
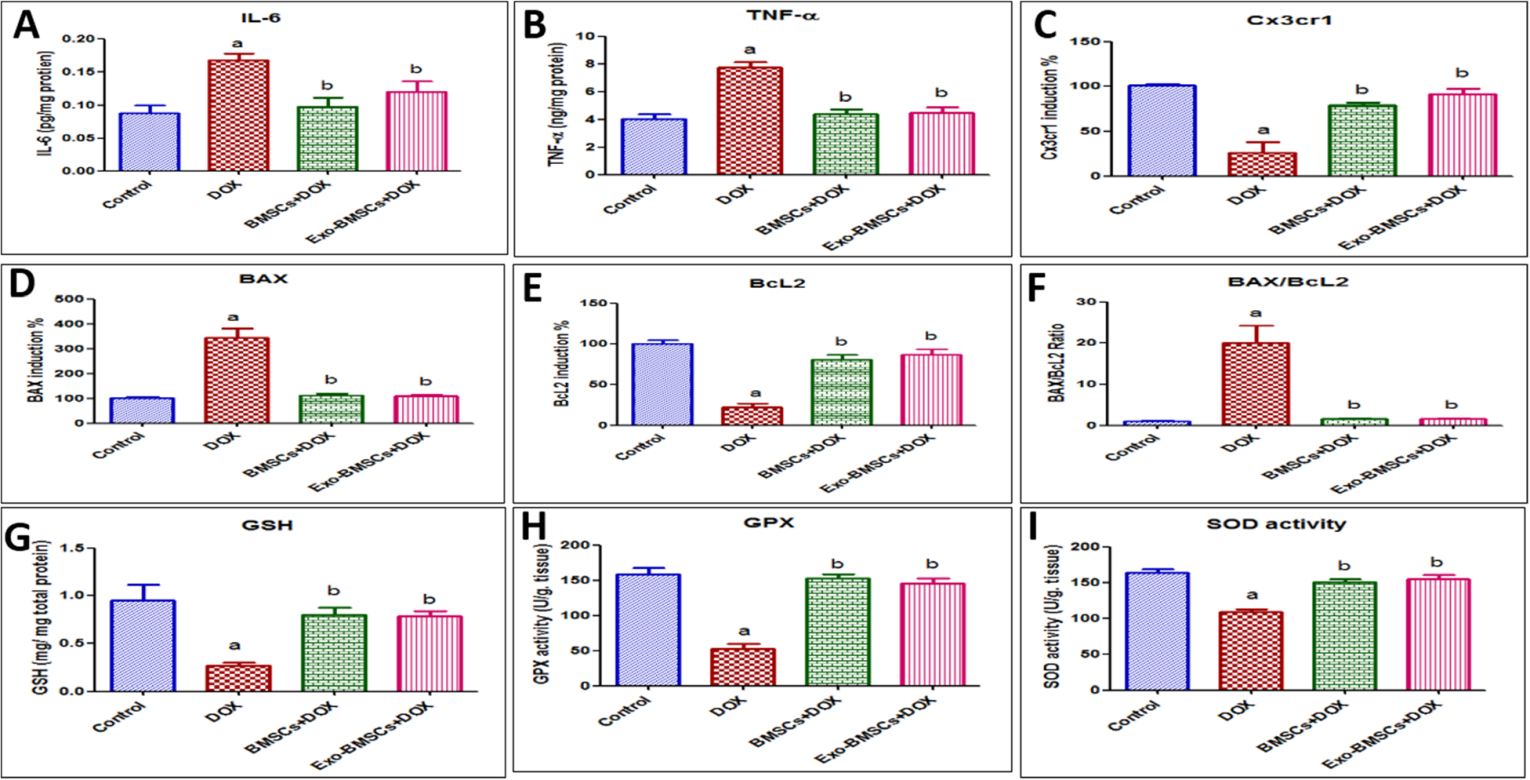
Effects of bone marrow stem cell (BMSC) and their exosome (BMSCs-Exo) treatment on inflammatory, apoptotic, and oxidative stress markers in doxorubicin (DOX)-induced chemobrain in rats. **A** A bar chart representing the protein expression of interleukin (IL)-6 in different rat groups. **B** A bar chart representing the protein expression of tumor necrosis factor (TNF)-α in different rat groups. **C** A bar chart representing the % of mRNA expression induction of Cx3cr1 in different rat groups. **D** A bar chart representing the % of mRNA expression induction of BAX in different rat groups. **E** A bar chart representing the % of mRNA expression induction of Bcl-2 in different rat groups. **F** A bar chart representing BAX/Bcl2 ratio in different rat groups. **G** A bar chart representing the abundance of glutathione (GSH) in brains of different rat groups. **H** A bar chart representing the activity of glutathione peroxidase enzyme (GPx) in brains of different rat groups. **I** A bar chart representing the activity of superoxide dismutase enzyme (SOD) in brains of different rat groups. Data are presented as mean ± SEM. Statistical Analysis was performed using one-way ANOVA followed by Tukey’s post hoc test (*n* = 6). ^a^ Significantly different from the control group at *p* < 0.05. ^b^ Significantly different from the DOX group at *p* < 0.05

Marked increase in the hippocampal inflammatory marker, IL-6, levels were found in DOX-induced chemobrain as compared to the control group. On the other hand, a significant decrease was detected in BMSC- and BMSCs-Exo-treated groups compared to the DOX-induced-chemobrain group as illustrated in Fig. 11A.

Similarly, there was a significant increase in the hippocampal TNF-α levels in DOX-induced chemobrain compared to the control group which was reversed in both the BMSCs and in the BMSCs-Exo-treated groups (Fig. 11B). Noteworthy, miR-21-5p was also found to regulate neuroinflammation through TNF and IL-6R targets as discovered by our IPA analysis.

The microglial fractalkine receptor, Cx3cr1, represent a primary neuron-microglia inter-regulatory system important for synaptic plasticity and function [52]. A significant decrease was found in Cx3cr1 gene expression in DOX-induced chemobrain as compared to the control group. Oppositely, Cx3cr1 gene expression was significantly increased in the BMSC- and the BMSCs-Exo-treated groups compared to the DOX-induced chemobrain group (Fig. 11C).

An apoptotic state was clearly demonstrated in the DOX-induced chemobrain group which is confirmed by a significant increase in BAX gene expression (Fig. 11D) and a significant decrease in Bcl2 gene expression (Fig. 11E) associated with a significant increase in BAX/Bcl2 ratio (Fig. 11F) compared to the control group. This apoptotic state was reversed upon treatment with BMSCs and BMSC-Exo as shown by a significant decrease in BAX gene expression and a significant increase in Bcl2 gene expression accompanied by a significant decrease in BAX/Bcl2 ratio compared to the DOX-induced chemobrain group (Fig. 11D–F).

### BMSCs and BMSCs-Exo reversed DOX-induced oxidative stress

Marked oxidative stress is one of the hallmarks of chemobrain [23]. In this context, a significant decrease in GSH levels was detected in DOX-induced chemobrain as compared to the control group **(**Fig. 11G), while BMSCs and BMSCs-Exo treatment successfully restored GSH levels as compared to the DOX-induced chemobrain group (Fig. 11G).

In alignment, the enzyme activity of GPx was significantly decreased in the DOX-induced chemobrain group as compared to control group while BMSCs and BMSCs-Exo significantly increased GPx activity compared to the DOX-induced chemobrain group (Fig. 11H). Additionally, our results show a significant decrease in SOD activity in the DOX-induced chemobrain group compared to the control group, which was abrogated by BMSCs or BMSCs-Exo treatments (Fig. 11I).

## Discussion

Since human postnatal neurodevelopment and cellular neuroplasticity are continuously ongoing, it is not surprisingly that neurological syndromes are well described across numerous populations treated with various chemotherapeutic regimens. This legitimate condition dramatically has a major negative impact on the patient’s quality of life [53]. Treating chemobrain is on the horizon, with much attention being drawn to it. However, no approved pharmacological intervention has yet been settled. In an attempt to find a protective approach against chemobrain with a commonly used cytotoxic agent, this study evaluated the effects of BMSCs and BMSCs-Exo in DOX-induced chemobrain.

Mounting studies reported that chronic exposure to clinically, cumulative relevant doses of DOX, a topoisomerase interactive agent, leads to unintended disruption of the hippocampus which ultimately ends by the development of chemobrain [54]. In this current study, we show that induction of chemobrain using DOX causes significant memory distortion confirmed by impaired learning and acquisition of short- and long-term spatial memory functions. Our findings were confirmed by a significant decrease in retention test time in PA test translating significant alterations in the contextual fear conditioning performance as well as a distorted short-term spatial memory in rats. Moreover, disturbed cognitive functions were also evidenced by a significant decrease in the percent of alteration observed in the Y-maze test. Interestingly, the special learning has been dramatically affected in the DOX-induced chemobrain rats. Furthermore, the mean escape latency failed to decrease in DOX-induced chemobrain rats throughout the whole experiment which was coupled with a significant decrease in the time spent in the platform quadrant in the probe tests for DOX-induced chemobrain. Collectively, these behavioral tests indicated failure in the development of spatial memory. This comes in alignment with previous studies that confirmed behavioral disturbance associated with DOX treatment in rats [55]. On the other hand, our model showed insignificant effect of DOX on the locomotor functions which agreed with previous reports as well [55].

Ameliorative effects of BMSCs and BMSCs-Exo were previously assessed in different cognitive deficits models [29, 30, 56]. Increasing evidences proved that BMSCs can successfully pass the blood-brain barrier (BBB) to reduce the injured brain area which subsequently leads to behavioral improvement [57]. This agrees with our results which showed successful homing of BMSCs within the brain hippocampal area. Besides, BMSC- and BMSCs-Exo-treated rats showed marked improvements in learning and acquisition of spatial memory functions besides significant restoring of the contextual fear performance in these rats as shown in various behavioral tests. Our findings align with evidences reported previously for the beneficial effects for BMSCs and their derived exosomes on behavior in different cognitive impairment diseases [58, 59].

Histologically, neurodegeneration was significantly confirmed upon DOX administration as explored by diffused neural damage and pyknosis in the hippocampal area in DOX-induced chemobrain. Furthermore, decreased myelin stain was significantly associated with DOX-induced chemobrain rats. In fact, neural myelination and disruption of oligodendrocyte lineage dynamics were reported previously to be influenced directly or indirectly via aggravated inflammation induced by DOX and its metabolites [60, 61]. This was strongly confirmed in our results with marked decreases in Olig2 and Opalin expression in the DOX-induced chemobrain groups, whereas Olig2 is an oligodendrocyte transcription factor which regulates oligodendrocyte differentiation promoting neural myelination [62] while Opalin is a CNS-specific myelin protein secreted by oligodendrocytes used to increase neural myelination [63].

Alongside this, a significant decrease in neurotropic growth factors such as BDNF and FGF-2 were also detected in DOX-induced chemobrain groups. As such, they play a major role in neural development and myelination [64, 65]. Additionally, our model of DOX-induced chemobrain was associated with disturbed synaptic markers where it revealed decreased gene and protein expressions of Syp. Furthermore, functional cholinergic transmitter was also significantly affected in our DOX-induced chemobrain model as confirmed by increased acetylcholinesterase activity which aligns with a previous study on chemobrain [23].

Beneficially, restoring histological hippocampal circuits was successfully attained upon BMSCs and BMSCs-Exo treatment in DOX-induced chemobrain. Cooperatively, neural re-myelination was observed in those groups which was evidenced by significant increases in Olig2 and Opalin expressions. In consistency, previous reports assured that MSCs successfully induce neural progenitor cell differentiation into oligodendrocytes which facilitates neural myelination via neurotrophic factor secretion. Besides, MSCs could promote increased expression of BDNF and other nerve growth factors which induce neural re-myelination in neurons [66]. Interestingly, treated groups with BMSCs and BMSCs-Exo significantly increased FGF-2 production. FGF-2 is believed to induce potent neurogenic effects which subsequently alleviate hippocampal neural apoptosis and modulate neurogenesis [67]. Furthermore, synaptic factors and functional neurotransmitters were successfully restored upon BMSC and BMSCs-Exo treatment. These findings agree with previous reports showing a significant increase in Syp after stem cell treatment in various neurodegenerative diseases [68, 69]. Besides, it was reported that successful targeting the cholinergic system was achieved using stem cell therapies in multiple cognitive deficits disorders [70] which agrees with our results of a significant decrease in acetylcholinesterase activity upon BMSC and BMSCs-Exo treatment.

These regenerative properties of BMSCs could be explained by direct differentiation or via secretion of neurotropic factors by BMSCs and their derived exosomes which indirectly stimulate the functional neuronal niche to secrete various neurotrophic factors and cytokines. Furthermore, exosomes are also known to be enriched by multiple bioactive reparative molecules such as miRNAs and others that could epigenetically modulate the expression of signaling molecules involved in neural regeneration pathways. Besides, these secreted bioactive molecules can attain anti-inflammatory, anti-apoptotic, and immunomodulatory effects which promote neural regeneration and activate the hippocampal circuits [66].

Regarding signaling pathways controlling neurodegeneration in DOX-induced chemobrain, our results show a significant decrease in the expression of various signaling molecules controlling the sonic hedgehog ligand, Shh, and its receptor, Ptch1, together with its transcription factor, Gli-1. It was reported previously that the hedgehog pathway, through Shh, controls the early stages of neurogenesis via activation of hippocampal progenitor cell proliferation and differentiation into neurons [11]. Early stage of differentiation is also aided by the canonical Wnt/β-catenin pathway [11]. Our results show a significant decrease in genes expression of Wnt-3a, Wnt-7b, FZD1, and β-catenin coupled with a significant decrease in growth factor (BDNF and FGF-2) and transcription factor expression (Sox2, NeuroD1, and Prox1). All these signals converge for structural integrity of hippocampal neurons and their generation from the adult neural stem cells pool with the subsequent integration into the functional hippocampal circuits [11].

Treatment with BMSCs or their derived exosomes succeeded in rectifying the imbalance in these pathways. Mechanistically, our study show that exosomes contain a miRNA-rich cargo which epigenetically modulate the expression of signaling molecules involved in the integrated Wnt/β-catenin and hedgehog signaling pathways. These integration results ultimately in restoring the disrupted key factors in Wnt/β-catenin and hedgehog signaling pathways as Wnt-3a, Wnt-7b, FZD1, β-catenin, Shh, Ptch1, and Gli-1. Accompanied by a significant increase in growth factor (BDNF and FGF-2) and transcription factor expression (Sox2, NeuroD1, and Prox1) coupled with a significant increase in NeuN gene expression. NeuN is a neuronal nuclear antigen, widely expressed in mature neurons as it regulates adult hippocampal neurogenesis and synaptogenesis [71].

Simultaneously, neurodegeneration is intricately associated with microglial activation which elicits an aggravated hippocampal inflammatory state. Microglia, the immune cells of the brain, are supposed to be neuroprotective; however, they can become neurotoxic following excessive or chronic activation. Microglial activation takes place by proinflammatory cytokines as TNF-α [72]. Our results confirmed the state of microglial activation by increased expression of IBA-1. Being a 17-kDa actin-binding protein, IBA-1 is specifically and constitutively expressed in activated microglia [73]. In line with our results, previous reports confirmed a significant increase in IBA-1 expression in chemotherapy-induced chemobrain [60]. Furthermore, it was proved that microglial activation leads to persistent astrocyte reactivation which is associated with long-term deficits in some neurological diseases [74]. These findings are consistent with our results with a significant increase in GFAP expression in DOX-induced chemobrain.

Interestingly, our results show a marked decrease in Cx3cr1 expression in DOX-induced chemobrain. It was previously proven that Cx3cr1 deletion is associated with chronic deterioration of the CNS injury. This was explained by microglial overactivation and overexpression of IL-1β and TNF-α with the lack of fractalkine Cx3cl1/Cx3cr1 signals [75]. In fact, Cx3cr1 receptors are reported to be expressed on the microglia, besides, its ligand, Cx3cl1, can act directly on neurons through Cx3cr1 receptors as well, to activate different neuronal survival pathways [76]. This suggests that Cx3cl1/Cx3cr1 signaling is involved in a complex network of interactions between neurons and glia [77].

In a positive feedback loop mechanism, the activated microglia and astrocytes in chemobrain concomitantly exaggerate the secretion of proinflammatory cytokines IL-6, IL-1β, and TNF-α. This inflammatory circuit directly impairs neural stem cell functions in the brain [78]. This state was asserted by a significant increase in the inflammatory cytokines such as IL-6 and TNF-α levels in the hippocampus of DOX-induced chemobrain. This inflammatory state is coupled with a generalized oxidative stress state provoked by DOX due to increased generation of intracellular reactive oxygen species (ROS) [79]. Ultimately, this state results in increased levels of protein oxidation as well as lipid peroxidation in brain neurogenic niche [80]. This was clearly observed in our model which showed a marked decrease in the antioxidant levels of GSH and its peroxidase enzyme GPx and in SOD activity which were reversed upon treatment with BMSCs and BMSCs-Exo. Additionally, our findings showed increased expression of pro-apoptotic factors in hippocampus which was assessed by increased Bax/Bcl2 ratio in response to oxidative stress in chemobrain which aligns with previous reports [23].

Beneficially, BMSCs and BMSCs-Exo successfully revoked this activated immune-inflammatory state with its deleterious sequels in DOX-induced chemobrain via decreasing hippocampal IL-6 and TNF-α levels. This effect is suggested to be through the increased abundance of miR-21-5p in the exosomal BMSCs as discovered by our IPA analysis, which is known for its anti-inflammatory role as agreed by previous studies [81, 82]. This anti-inflammatory state leads to a significant decrease in the activated microglia in the brain and the activated astrocytes evidenced by a significant decrease in the IBA-1 and GFAP expression. These results are in accordance with previous reports which showed that BMSCs and BMSCs-Exo can modulate microglial activation in stroke [83]. These effects were accompanied by a significant increase in Cx3cr1 expression, which has been reported to attain neuroprotective and neuro-modulatory functions [76]. Therefore, increased expression of cx3cr1 in BMSC- and BMSCs-Exo-treated groups might be an indicator for neuroprotective effects which need further elucidation.

Moreover, it was documented previously that MSCs can potentially manage an oxidative stress state due to increased expression of antioxidant enzymes as GPx and catalase [84]. This agrees with our findings of increasing GSH levels and GPx, SOD activity in BMSC- and BMSCs-Exo-treated rats. This antioxidant effect of BMSCs and BMSCs-Exo potentially enforces their anti-inflammatory properties in our treated groups.

Recent attention has been drawn to some disadvantages of MSCs which includes possible immune rejection to allograft transplantation, uncontrollable quality, and potential tumorigenicity [85]. Interestingly, our study concluded that BMSCs-Exo had a comparable effect to BMSCs against DOX-induced chemobrain and thus exosomes are expected to replace stem cells for tissue repair as a safe therapeutic tool.

## Conclusion

Restoring structural hippocampal circuits has become an indispensable need among cancer survivors. Promising results for BMSC- and BMSC-derived exosomes against DOX-induced chemobrain are introduced. Our study identified some BMSCs secreted exosomal miRNAs that could potentially restore the balance in Wnt/β-catenin and hedgehog signaling pathways and resolve the complex inflammatory, oxidative, and immune crosstalk in chemobrain which ultimately pave the road for the restoration of healthy hippocampal circuits in adult chemobrain. Future mechanistic studies are warranted to delineate this genetic and epigenetic integration which could potentially participate in understanding and treating doxorubicin-induced cognitive impairment.

## Supplementary Information

**Additional file 1: Suppl. Fig. 1.** (A) A network reflecting the connection between the discovered miRNA in BMSC-Exo and sporadic amyotrophic lateral sclerosis. (B) A network reflecting the connection between the discovered miRNA in BMSC-Exo and amyotrophic lateral sclerosis. (C) A network reflecting the connection between the discovered miRNA in BMSC-Exo and progressive motor neuropathy. (D) A table showing the disease categories associated with the most abundant miRNA in BMSC exosomes.

**Additional file 2.**

**Additional file 3.**

## Data Availability

The data that support the findings of this study are available from the corresponding author upon reasonable request. Some data may not be made available because of privacy or ethical restrictions.

## Abbreviations

BMSCs: Bone marrow mesenchymal stem cells
BMSCs-Exo: BMSC-derived exosomes
DOX: Doxorubicin
Shh: Sonic hedgehog
LDMEM: Low-glucose Dulbecco’s modified Eagle’s medium
TAE: Total arm entries
SAP: Spontaneous alternation percentage
Olig2: Oligodendrocyte transcription factor-2
SY 38: Synaptophysin
Opalin: Oligodendrocytic paranodal loop protein
qRT-PCR: Quantitative real-time PCR
BCA: Bicinchoninic acid
BDNF: Brain-derived neurotropic factor
FGF-2: Fibroblast growth factor-2
Wnt: Wingless-type MMTV integration site family
β-catenin: Beta-catenin
Neurod1: Neurogenic differentiation 1
Prox1: Prospero-related homeobox
NeuN: Fox-3, Rbfox3, hexaribonucleotide binding protein-3
SOX2: Sex-determining region Y-box 2
Sry: Vimentin, sex-determining region Y
AChE: Acetylcholinesterase
GFAP: Glial fibrillary acidic protein
Iba1: Ionized calcium-binding adaptor molecule 1
TNF-α: Tumor necrosis factor-α
IL-6: Interleukin-6
Cx3cr1: CX3C chemokine receptor 1
Bcl2: B cell lymphoma 2
BAX: BCL2-associated X
ANOVA: Analysis of variance
