# Supplementary figures and images for: Bone marrow mesenchymal stem cells and their derived exosomes resolve doxorubicin-induced chemobrain: critical role of their miRNA cargo
Source: Stem Cell Res Ther. 2021 Jun 5;12:322. doi: 10.1186/s13287-021-02384-9 (PMC8180158; doi:10.1186/s13287-021-02384-9)

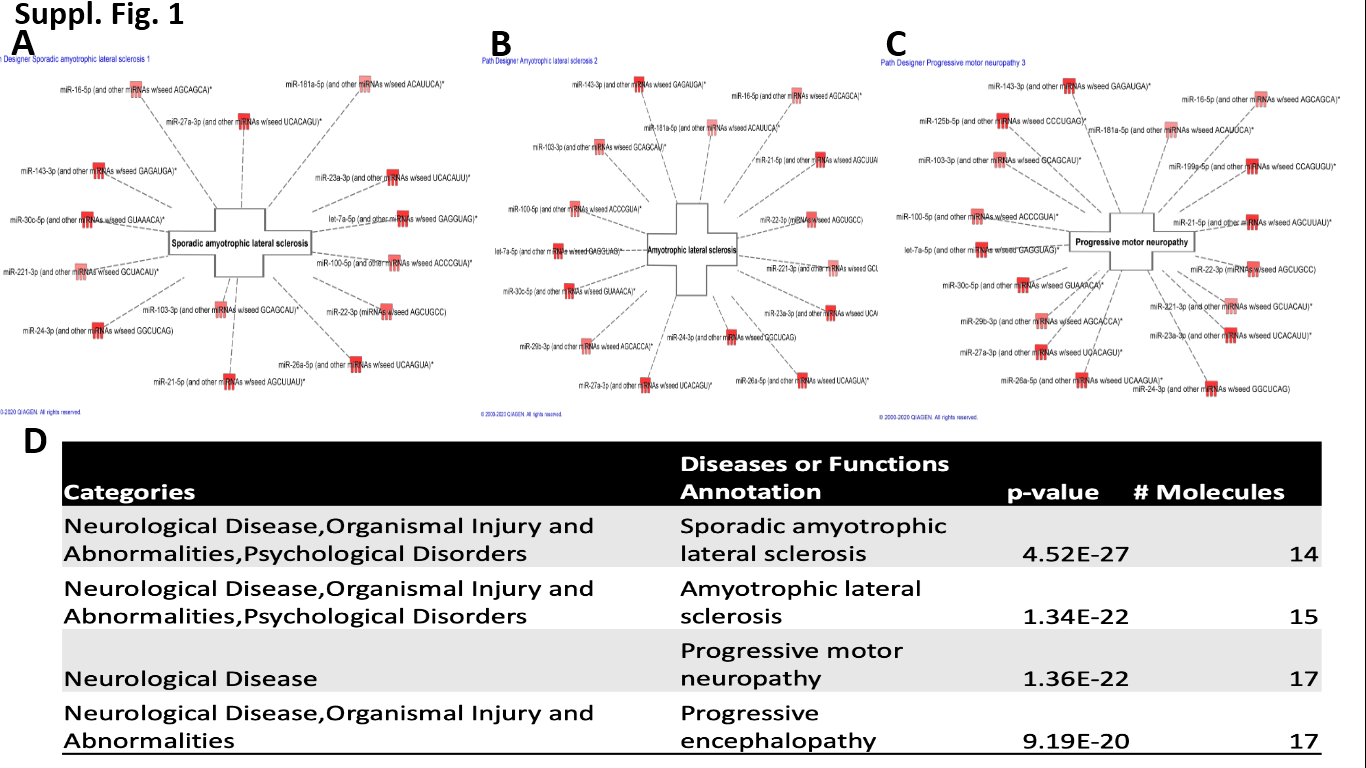

Supplement: Supplementary file 1 — Additional file 1: Suppl. Fig. 1. (A) A network reflecting the connection between the discovered miRNA in BMSC-Exo and sporadic amyotrophic lateral sclerosis. (B) A network reflecting the connection between the discovered miRNA in BMSC-Exo and amyotrophic lateral sclerosis. (C) A network reflecting the connection between the discovered miRNA in BMSC-Exo and progressive motor neuropathy. (D) A table showing the disease categories associated with the most abundant miRNA in BMSC exosomes. [file 13287_2021_2384_MOESM1_ESM.png]
